# 2D Layers of Group VA Semiconductors: Fundamental Properties and Potential Applications

**DOI:** 10.1002/advs.202203956

**Published:** 2022-10-26

**Authors:** Saima Batool, Muhammad Idrees, Su‐Ting Han, Ye Zhou

**Affiliations:** ^1^ Institute for Advanced Study Shenzhen University Shenzhen 518060 P. R. China; ^2^ Additive Manufacturing Institute College of Mechatronics and Control Engineering Shenzhen University Shenzhen 518060 P. R. China; ^3^ College of Electronics Science & Technology Shenzhen University Shenzhen 518060 P. R. China

**Keywords:** 2D group VA materials, electronics, monoelemental semiconductor allotropes, optoelectronics, synthetic approaches

## Abstract

Members of the 2D group VA semiconductors (phosphorene, arsenene, antimonene, and bismuthine) present a new class of 2D materials, which are recently gaining a lot of research interest. These materials possess layered morphology, tunable direct bandgap, high charge carrier mobility, high stability, unique in‐plane anisotropy, and negative Poisson's ratio. They prepare the ground for novel and multifunctional applications in electronics, optoelectronics, and batteries. The most recent analytical and empirical developments in the fundamental characteristics, fabrication techniques, and potential implementation of 2D group VA materials in this review, along with presenting insights and concerns for the field's future are analyzed.

## Graphical abstract

Members of the 2D group VA materials (phosphorene, arsenene, antimonene, and bismuthine) have presented their fascinating structures and intriguing electronic properties. The most recent analytical and empirical developments in the fundamental characteristics, fabrication techniques, and potential implementation of 2D group VA materials in this review, along with presenting insights and concerns for the field's future are analyzed.

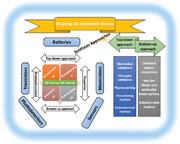

## 1 Introduction

2D materials have intriguing physiochemical characteristics and sparked substantial interest in various sectors, e.g., electronic and optoelectronic devices due to their large aspect ratios, quantum size effects, and mechanical flexibility.^[^ ^1^ ^]^ Members of 2D group VA members including phosphorene (P), arsenene (As), antimonene (Sb), and bismuthene (Bi) are particularly appealing for applications in upcoming nanoelectronic devices, which may result in reduced energy consumption.^[^ ^2^ ^]^ The most significant advantages of group VA semiconductors are their effective tunable direct bandgap (0.3 to 2.62 eV) and charge carrier mobility compared to transition metal dichalcogenide (TMD) materials and semimetallic group IVA materials.^[^ ^3^ ^]^ A variety of monolayered 2D group VA materials have been investigated and exhibited original/unique properties.^[^ ^4^ ^]^

Significantly, the most studied 2D group VA member is phosphorene, which exhibits a direct band gap with large tunability, from 0.3 eV in the bulk to 2.0 eV in monolayers.^[^ ^5^ ^]^ Phosphorene cousins (As, Sb, Bi) have also caught the attention of researchers due to their fascinating structures and interesting electronic properties. Members of the 2D group VA are expanding the scope of their applications in diverse fields, and have fascinated the scientific community of materials science, chemistry, and physics in the last few years.^[^ ^4^, ^6^ ^]^ Li and co‐workers^[^ ^2f^ ^]^ fabricated a few‐layered phosphorene‐based field‐effect transistor (FET) in 2014 which was acknowledged as having outstanding properties due to its extraordinary performance. Furthermore, phosphorene is a p‐type semiconductor with extremely high charge carrier mobility (up to 10 000 cm^−2^ V^−1^ s^−1^),^[^ ^2c^ ^]^ making it a good choice for semiconductor devices.^[^ ^7^ ^]^ In addition, phosphorene has good in‐plane anisotropy which is another key benefit.^[^ ^8^ ^]^ Scientists^[^ ^4^ ^]^ predicted single‐layered arsenene and antimonene with the characteristics of tunable direct bandgap and high charge carrier mobility with preferable stability based on the efficient synthesis of phosphorene. Moreover, arsenene and antimonene are typically semimetals in bulk. When thinned to one atomic layer, they transform into indirect semiconductors with bandgaps of 2.49 and 2.28 eV, respectively.^[^ ^4a^ ^]^ Following innovative fabrications of single‐layer and few‐layer group VA materials by utilizing multiple techniques, significant research preferences have focused on 2D group VA components. A few group VA materials and their hybrid heterostructure have also been investigated by incorporating one group VA element with another group VA element, or another element. The above mention 2D compounds exhibit variable bandgaps associated with diverse components, making the intriguing choices for photonic and microelectronic applications.

To synthesize the 2D group VA layered materials, researchers have used various approaches such as mechanical exfoliation, vapor deposition techniques, liquid‐phase exfoliation, molecular beam epitaxy, and plasma‐assisted methods.^[^ ^9^ ^]^ Meanwhile, researchers have reported on the appealing diverse variety of bandgaps of group VA elements using extensive density functional theory (DFT) simulations.^[^ ^1c^ ^]^ The diverse bandgaps are beneficial for broad‐spectrum photoresponse.^[^ ^7^ ^]^ 2D group VA monolayers come in a variety of allotropes. The *α*‐phosphorene, *α*‐bismuthene, and *β*‐bismuthene allotropic semiconductors have bandgaps of 1.83, 0.36, and 0.99 eV, correspondingly.^[^ ^10^ ^]^ On the other hand, *α*‐arsenene and *α*‐antimonene display 1.68 and 1.43 eV quasi‐direct bandgaps, respectively. In addition, the diverse energy levels (conduction bands minimum and valence band maximum) of 2D group VA single‐layered elements may make it easier to fabricate 2D heterostructures that have exciting applications in the optoelectronic field.^[^ ^11^ ^]^

**Figure 1:**
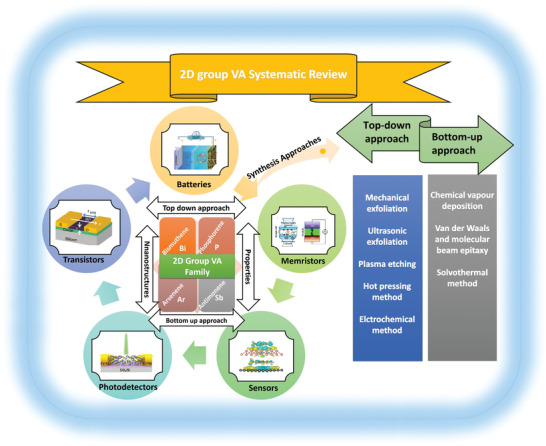
A diagram illustrates the major ideas of the whole review as well as the wide variety of 2D group VA materials, their synthesizing strategies, structural components, and implementations. Memristors: Reproduced with permission.^[^ ^112^ ^]^ Copyright 2019, Royal Society of Chemistry. Sensors: Reproduced with permission.^[^ ^109^ ^]^ Copyright 2019, Springer Nature. Photodetectors: Reproduced with permission.^[^ ^80^ ^]^ Copyright 2020, Wiley‐VCH. Transistors: Reproduced with permission.^[^ ^3b^ ^]^ Copyright 2014, Springer Nature. Batteries: Reproduced with permission.^[^ ^111a^ ^]^ Copyright 2017, Wiley‐VCH.

It is believed that the unique 2D group VA materials would accompany the features of low‐dimensional materials, thereby establishing a novel research area in the ground of nanostructured materials. Researchers have widely studied the chemical and physical properties of elemental 2D materials. The constantly rising number of papers in the field of 2D group VA materials offers dynamic inspiration for this comprehensive review. Following an overview of 2D layers of group VA semiconductors, this article discusses the synthesis processes and characteristics of phosphorene, arsenene, antimonene, and bismuthene for applications in many fields. Several top‐down and bottom‐up approaches are summarized. We then emphasize their electrical, electrochemical, mechanical, and thermal characteristics (**Figure** **1**). Furthermore, numerous functional nanostructures based on 2D group VA layered materials are explored, including various heterostructures and functionalized nanostructures. A wide range of exciting applications has been discussed, encompassing several important sectors, for instance, FETs, photodetectors, memristors, sensors, and batteries. Finally, we address the overall perspective for the potential applications, including the problems and prospects.

This timely and comprehensive review article discusses the most recent and interesting developments in the 2D layered group VA monoelemental materials, which can attract international scientific societies of material science^[^ ^11^, ^12^ ^]^ and device engineering.^[^ ^13^ ^]^ The analysis will assist in the development of 2D layered materials including monoelemental single‐layered, multi‐layered, hybrid, and functionalized low‐dimensional materials.

## 2 2D Group VA Semiconductor Allotropes with High Carrier Mobilities

**Figure 2:**
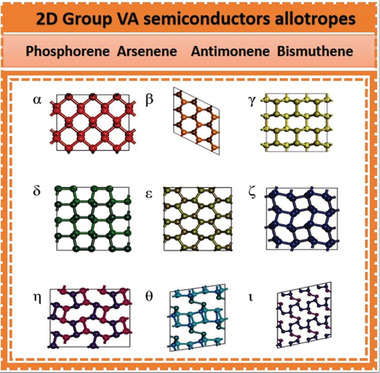
Uppermost interpretations of relaxed 2D Group VA allotropes which have conventional five “honeycomb shaped” structures (*α*, *β*, *γ*, *δ*, ɛ) and four “non‐honeycomb” structures (*ζ*, *η*, *θ*, *ι*). Reproduced with permission.^[^ ^4b^ ^]^ Copyright 2015, Wiley‐VCH.

**Figure 3:**
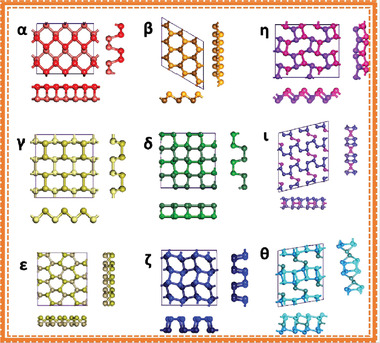
Relaxed group VA monolayer allotropes: topmost interpretations and sideways interpretations (x and y horizontal directions) thru five conventional honeycombs' structural chemistry (*α*, *β*, *γ*, *δ*, *ε*). Moreover, four non‐honeycomb structural chemistry (*ζ*, *η*, *θ*, *ι*). Reproduced with permission.^[^ ^4b^ ^]^ Copyright 2015, Wiley‐VCH and Sons.

**Figure 4:**
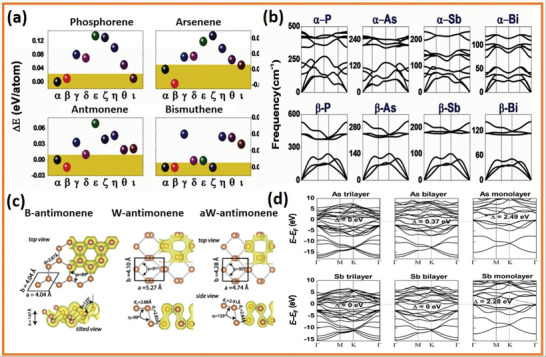
a) The 2D group VA member's average binding energy. b) Diffusions of phonon bands that are thermodynamically stable. Reproduced with permission.^[^ ^4b^ ^]^ Copyright 2015, Wiley‐VCH. c) 2D single‐layer structures of antimony. Reproduced with permission.^[^ ^29^ ^]^ Copyright 2015, American Physical Society. d) Evaluation of (tri, bi, and monolayers of arsenene and antimonene) electrical band structures on HSE06 Fermi levels. Reproduced with permission.^[^ ^3a^ ^]^ Copyright 2015, Wiley‐VCH.

**Figure 5:**
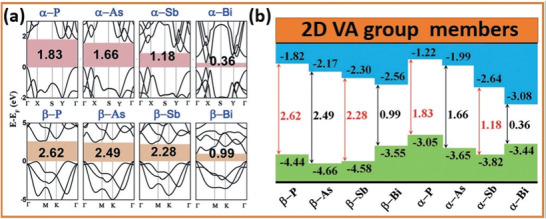
Investigations of electronic properties (via HSE06 level of theory). b) Wideband photo response of 2D VA group semiconductors. Reproduced with permission.^[^ ^4b^ ^]^ Copyright 2015, Wiley‐VCH.

2D group VA elements have various structural differences, including *α*, *β*, *γ*, *δ*, and *ɛ* with distinctive honeycomb‐like structures as well as *ζ*, *η*, *θ*, and *ι* with non‐honeycomb like structures, as shown in **Figures** **2** and **3**. The average binding energies [*E* (eV per atom)], the lattice constants [*a* and *b*], and the thickness [*h*] of group VA monolayers are presented in **Table** **1**. The phosphorene with puckered structure presents the lowermost binding energy among the following allotropic configurations of *α*, *β*, *γ*, *δ*, *ɛ*, *ζ*, *η*, *θ*, and *ι*, which is in full correspondence with earlier findings. Nevertheless, *β* phases with their buckling forms are proved to be the utmost stable among many allotropes (arsenene, antimonene, and bismuthene allotropes), which deviate from the lowest‐energy configuration of phosphorene (**Figure** **4**a). At the same theoretical level, three phases of bismuthine (*α*, *β*, and *ζ*) exhibit equal average binding energies. Under standard conditions, bulk‐type materials of the single‐layer *α*‐phosphorene (*α*‐P) are black phosphorus (BP). It is the most stable allotrope in all other modified forms, e.g., white, black and red phosphorus and some amorphous forms. Bulk rhombohedral gray color arsenic, antimony, and bismuth resemble to *β* allotropic forms of 2D group VA members (arsenene, antimonene, and bismuthine) in the presence of typical conditions through the similar space group. Moreover, the *β*‐bismuth, as well as gray arsenic, have the same crystal structural morphology. Considering the energetic stability and successful fabrication in the experiment, we mainly focus on the *α* and *β* phases of single‐layered 2D group VA materials (*α*‐P, *α*‐As, *β*‐As, *β*‐Sb, and *β*‐Bi).^[^ ^14^ ^]^ The phonon spectra of these free‐standing 2D group VA materials should also be analyzed, which also behave as a basis for evaluating thermodynamic consistency. Figure 4b shows that 2D group VA single elemental layers are available. Therefore, they have no discernible fictitious phonon modes, proving that they are thermodynamically stable. Bulk crystals of *α*‐P and *β*‐P, As, Sb, and P are anticipated to exfoliate easily to form a 2D monolayer owing to the weak interlayer interactions. *α*‐phosphorene can be productively exfoliated from bulk *α*‐P, and *β*‐bismuthene can also be grown on diverse substrates. Antimony single‐layer frameworks are presented in Figure 4c, and *β*‐antimonene has buckled honeycomb structure with a hexagonal lattice. Structure parameters such as lattice constants, bond lengths, and bond angles are also shown with optimized values. The isosurfaces and contour plots of the total charge density show the bonding between Sb atoms which is the buckling, in which Sb atoms on the hexagon's corners alternately move up and down. The same is true for a 2D and symmetric washboard structure, such as W‐antimonene, which has a 2D rectangular lattice. The other 2D group VA single‐layers can be experimentally fabricated soon for the recent quick application of assembling 2D materials. **Figure** **5**a displays the energy levels of group VA members’ free‐standing (*α* and *β*) phases. The bandgaps of *α*‐phases of 2D group VA member's monolayers are less than those of the corresponding monolayers of *β*‐phases. *α*‐phosphorene (monolayers), *α* and *β*‐bismuthene (monolayers) have direct bandgap values of 1.83, 0.36, and 0.99 eV, correspondingly. Additionally, at the Γ point, *α*‐arsenene and *α*‐antimonene exhibit “quasi‐direct bandgaps” of 1.68 and 1.43 eV, respectively. The rest are semiconductors with indirect bandgaps. These findings point to the conclusion that the covalent properties will progressively deteriorate for heavier VA atoms.

**Table 1:** Energetics and structural parameters of all group VA monolayer allotropes: Δ*E* (eV per atom) are the average binding energies; *a* and *b* are the lattice parameters; *h* denotes the thickness of the group VA monolayers

Models	Δ*E* [eV per atom]	*a* [Å]	*b* [Å]	*h* [Å]
P‐*α*	0.00	4.53	3.36	
P‐*β*	0.01	3.33	3.33	
P‐*γ*	0.08	3.41	5.34	
P‐*δ*	0.07	5.56	5.46	
P‐*ε*	0.135	5.78	6.21	
P‐*ζ*	0.13	5.37	5.37	
P‐*η*	0.10	6.43	5.32	
P‐*θ*	0.05	5.40	6.32	
P‐I	0.01	5.50	6.22	
As‐*α*	0.000	3.69	4.76	2.40
As‐*β*	‐0.037	3.61	3.61	1.40
As‐*γ*	0.032	3.59	5.90	1.69
As‐*δ*	0.034	5.91	5.94	2.42
As‐*ε*	0.072	6.34	6.80	1.71
As‐*ζ*	0.087	5.84	5.84	2.42
As‐*η*	0.054	7.09	5.85	2.26
As‐*θ*	0.025	5.93	7.01	3.96
As‐I	0.011	6.03	6.96	3.87
Sb‐*α*	0.000	4.35	4.89	2.82
Sb‐*β*	‐0.014	4.12	4.12	1.65
Sb‐*γ*	0.033	4.10	6.62	1.99
Sb‐*δ*	0.010	6.34	6.55	2.84
Sb‐*ε*	0.069	7.22	7.64	2.02
Sb‐*ζ*	0.039	6.42	6.37	3.20
Sb‐*η*	0.046	8.09	6.53	2.61
Sb‐*θ*	0.019	6.53	7.86	4.66
Bi‐*α*	0.000	4.59	5.01	2.99
Bi‐*β*	0.000	4.34	4.34	1.73
Bi‐*γ*	0.040	4.31	6.89	2.11

Various electronic characteristics might be caused by slight structural variations. Significantly, the bandgaps of 2D group VA monolayers are appropriate for prospective electronic/optoelectronic applications. The outcomes of the effective performance measurement show that the bandgaps vary widely from 0.36 to 2.62 eV. The photon frequency associated with the 2.62 eV bandgap falls within the range of blue light. In addition, the absorption coefficient is also crucial for optoelectronic applications. Figure 5b presents the wideband photoresponse of the 2D group VA semiconductors.

**Figure 6:**
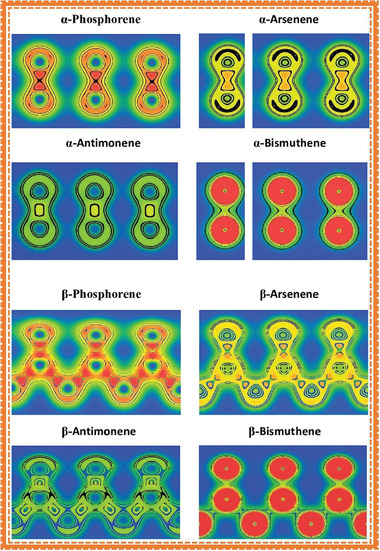
Slices of electron density for *α* and *β* phases of group VA monolayers. Reproduced with permission.^[^ ^4b^ ^]^ Copyright 2015, Wiley‐VCH.

**Table** **2** displays the optimal structural parameters. There are two different linkages for group VA monolayers in the *α*‐phase, *V*_Ai_—*V*_Ai_, and *V*_Ai_—*V*_Ao_, whose bond lengths gradually increase with the increase of atomic radius. There is only one type of bond in the *β*‐phase of group VA monolayers, and the bond lengths increase from 2.27 to 2.98 Å in the case of *β*‐phosphorene and *β*‐bismuthene. Moreover, there is an important shift between *α*‐phosphorene and some other *α*‐phases of group VA monolayers. In *α*‐phosphorene, the Pi—Po bond length is 2.25, which is longer than the Pi—Pi bond length (2.22). In other *α*‐phases of group VA monolayers, the *V*_Ai_—*V*_Ao_ bond length is shorter than the *V*_Ai_—*V*_Ai_, as presented in Table 2. The slight differences in structure between *α*‐P and other *α*‐phases of group VA monolayers may influence the outcome in different electronic properties. Furthermore, researchers have estimated the overlap populations and charge density as presented in **Figure** **6**.

**Table 2:** Mulliken overlap populations of the bonds and their bond lengths of group VA monolayers

VAene models	Bond	Population	Length [Å]
*α*‐Phosphorene	Pi–Po/Pi–Pi	0.38/0.84	2.247/2.222
*α*‐Arsenene	Asi–Aso/Asi–Asi	1.62/1.00	2.429/2.440
*α*‐Antimonene	Sbi–Sbo/Sbi–Sbi	1.01/0.37	2.725/2.748
*β*‐Phosphorene	P–P	1.03	2.267
*β*‐Arsenene	As–As	1.21	2.453
*β*‐Antimonene	Sb–Sb	1.32	2.755
*β*‐Bismuthene	Bi–Bi	1.15	2.979

Throughout the past ten years, 2D materials have appeared as promising candidates for photonic and optoelectronic applications.^[^ ^7^ ^]^ Graphene has extremely high mobility and a strong relationship with light at wavelengths ranging from microwave to ultraviolet, empowering various applications such as conductive films, optical modulators, photodetectors, and touch screen devices.^[^ ^8a^ ^]^ However, graphene‐based light‐emitting devices are impossible due to the zero bandgap. On the other hand, the carrier mobilities of 2D TMDs are several orders of magnitude lower than graphene's, leading to slow speeds.^[^ ^15^ ^]^ For advanced electronic and optoelectronic applications, novel 2D materials with high charge carrier mobilities and adequate electronic bandgaps are needed. Group VA semiconductors have shown acceptable bandgaps with high charge carrier mobilities, which are eagerly anticipated for optoelectronic applications.^[^ ^4b^ ^]^ Phosphorene, arsenene, and bismuthene have high charge carrier mobilities of thousands of cm^2^ V^−1^ s^−1^ range.^[^ ^16^ ^]^ For instance, phosphorene holds high charge carrier mobility of 10^3^ to 10^4^ cm^2^ V^−1^ s^−1^ with a 2.0 eV bandgap.^[^ ^17^ ^]^ Usually, *α*‐phases of 2D group VA monolayers have higher values of carrier mobilities than those of *β*‐phases.^[^ ^18^ ^]^ Furthermore, it is noteworthy that monoelemental *α*‐arsenene has higher mobility (up to 10^4^ cm^2^ V^−1^ s^−1^) than III‐V semiconducting materials.^[^ ^4b^ ^]^ In addition to having a comparatively tiny efficient electron mass of 0.21, *α*‐arsenene also shows a very low deflection potential of 0.65 eV, contributing to its high carrier mobility (**Table** **3**).

**Table 3:** Synthesis approaches, thickness, sources, and characteristics of 2D group VA nanomaterials

2D nanomaterials	Preparation method	Thickness	Precursor source	Characteristics	Refs.
Quasi 2D Bi_2_Te_3_	Mechanical exfoliation	Layered	Bulk Bi_2_Te_3_	Topological insulators, quasi‐2D crystals	[57]
2D K_3_BiAs_6_Se_12_	Solvothermal reaction	Layered	Bi powder	Be expected applying fluxes	[114]
Bi_2_S_3_ nanosheets	Probe, bath sonication, LPE	4–9 layers, 4.2–9.9 nm	Bi_2_S_3_ powder	High‐performance photodetectors	[115]
Bi_2_Te_3_ nanoplates	Solvothermal, vapor phase growth	Layered	Bi_2_Te_3_ powder	Optical transmission enhancement	[116]
Antimonene	Micromechanical exfoliation, LPE	Layered	Sb flakes	Noncovalent functionalization	[117]
Antimonene	LPE	Few‐layer, 4–20 nm	Layered Sb bulk	Nonlinear all‐optical signal processing	[118]
Antimonene	Micromechanical exfoliation, transfer	Bilayers, ≈1.8 nm	Sb material	Mechanical isolation, high stability	[119]
Antimonene	Electrochemical exfoliation	Multilayers, 31.6 nm	Bulk Sb	Broadband nonlinear optical response	[78]
Antimonene	Epitaxial growth on Ag(111)	1 ML, 2.2 Å	Sb atoms	Flat ML film with honeycomb structure	[120]
Antimonene	Sb deposition on Bi nanoislands	1–4 ML	Sb atoms	Engineering multiple topological phase	[121]
Arsenene	Plasma‐assisted process on InAs	Multilayers, ≈14 nm	InAs substrate	Multilayer arsenene nanoribbons	[122]
Phosphorene	Mechanical exfoliation	Mono to multilayer	P atoms	Field effect transistor	[2f]
Phosphorene	Liquid phase exfoliation	Mono to multilayer	P atoms	Biomedical	[69]
Phosphorene	Electrochemical exfoliation	Few‐layer	P atoms	–	[123]
Graphene‐PB heterostructure	Chemical vapor deposition	Few‐layer	PB bulk	Heterostructure for photo‐detection	[102]
BiQDs	Liquid phase exfoliation	4.9 nm	Bulk Bi	High‐performance photodetection	[64]
2D Sb_2_Se_3_	Chemical vapor deposition	1.3 nm	Sb_2_Se_3_	Photodetection	[80]
2D bismuth nanosheets	Liquid phase exfoliation	0.5–2 µm	Bulk bismuth	Photodetection	[124]
Sb_2_Se_3_ nanosheets	Hydrothermal method	Few‐layer	SbCl_3_	Photodetection	[125]
2D black phosphorus nanosheets	Liquid phase exfoliation	4 µm	Bulk BP	Photodetection	[100a]

In summary, the 2D group VA semiconductors may emerge as promising candidates for applications in microelectronics and optoelectronics owing to their outstanding carrier mobilities and various bandgaps. The current research on electronic structures and properties is believed to be significant in the development of 2D group VA materials.

## 3 Basic Properties of 2D Group VA Semiconductors

### 3.1 Phosphorene

The first and most researched participant of the 2D group VA family is phosphorene. Phosphorene seems to have a puckered framework with less symmetry than graphene, leading to two anisotropic in‐plane orientations. Appropriate project management is being made to unlock BP's maximum capabilities. Surprisingly, the recent high‐profile presentation of phosphorene (the monolayer of BP) has provided this component with a golden age. Hultgren and co‐workers^[^ ^19^ ^]^ ascertained the crystalline structure of BP for the first time in 1935 using X‐ray diffraction experiments on BP granules. It has intriguing properties such as highly adjustable bandgap, negative Poisson's ratio, high charge carrier mobility, and principally anisotropic electrical and optical features. It is generally regarded as among the most abundant on the planet (about 0.1 percent of an earth's crust).^[^ ^20^ ^]^ Interestingly, external factors such as heteroatom doping, defect introduction, electric field, strain, heterostructure synthesis, and chemical modification could be used to tune these characteristics.

**Figure 7:**
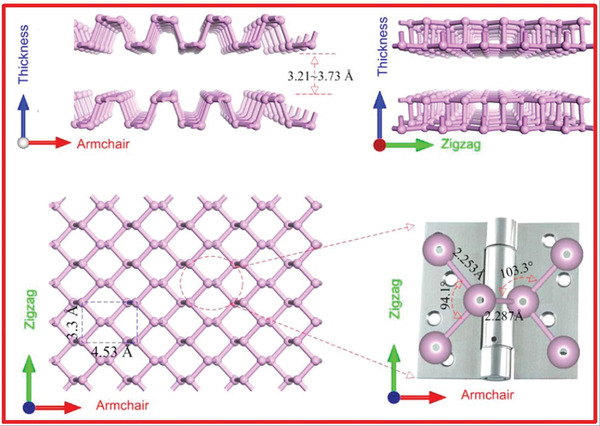
Armchair orientation and zoomed‐in local atomic framework of the phosphorus—phosphorus bond formation in an arrangement of phosphorene. Reproduced with permission.^[^ ^23^ ^]^ Copyright 2015, Wiley‐VCH.

Phosphorene, in particular, has efficient application benefits in electronic and optoelectronic devices. Nonetheless, phosphorene has an Achilles’ heel because of its rapid oxidation and light‐induced deterioration of exfoliated flakes in humid conditions. Several methods have been developed to address this problem, and the stabilization mechanisms have been investigated. Therefore, the practical implementation of phosphorene is in its adolescence. To hasten the commercialization of phosphorene‐based electronic devices, investigators must address the issue of oxidation and improve the material's long‐term stability. Since the successful exfoliation of graphene in 2004, mechanical exfoliation has been widely used to prepare 2D materials. Mono and few layer phosphorene were successfully fabricated and adopted for transistors in 2014 using the traditional exfoliation process. Immediate access to elemental phosphorus is challenging owing to its reactivity. Phosphorus can be categorized into four allotropes: red, white, violet, and black.^[^ ^21^ ^]^ White phosphorus, explored in 1669, has a tetrahedral structure. In contrast, red phosphorus has a chain‐like structure derived from the P4 tetrahedron in which a P—P bond (bond energy = 200 kJ mol^−1^) decomposes to form the new P—P bond only with the closest P4 tetrahedron as shown in **Figure** **7**. Violet phosphorus could be manufactured from red phosphorus through heating for a long time at 550 °C in the existence of liquid Pb.^[^ ^22^ ^]^ Comprehensive analytical and empirical research has found novel properties with intrinsic anisotropy. This distinguishing feature differentiates phosphorene from the rapidly growing family of 2D layered materials, holding great promise for electronics, photonics, and spintronics applications. The ongoing study usually concentrates on phosphorene applications and device demos based on its enticing bandgap and charge carrier mobility with highly anisotropic structural properties.^[^ ^23^ ^]^

### 3.2 Arsenene

**Figure 8:**
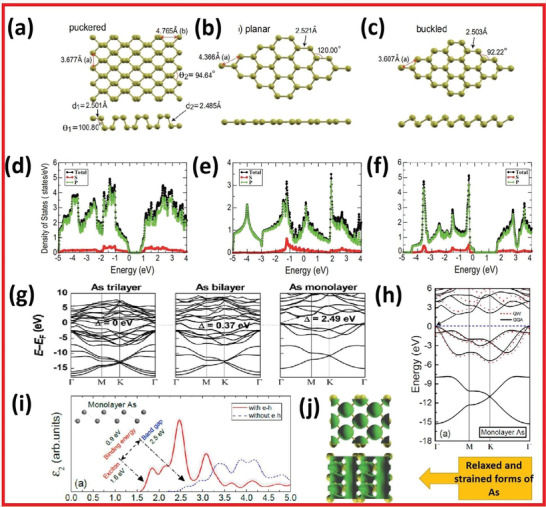
Fully optimized structure of a) puckered, b) planar, and c) buckled arsenene. Total and partial density of states of d) puckered, e) planar, and f) buckled arsenene. Reproduced with permission.^[^ ^18b^ ^]^ Copyright 2015, American Physical Society. g) Electronic band structures of monolayer, bilayer, and trilayer of buckled arsenene.^[^  Reproduced with permission.^[^ ^3a^ ^]^ Copyright 2015, Wiley‐VCH. h) Band structures of multilayered arsenene. i) Calculated imaginary part of the dielectric function *ε*_2_ for multi‐layered arsenene. Reproduced with permission.^[^ ^27^ ^]^ Copyright 2017, American Chemical Society. j) Relaxed and strained forms of As. Reproduced with permission.^[^ ^18b^ ^]^ Copyright 2015, American Physical Society.

Following the rediscovery of phosphorene in the 2D group VA semiconductors, 2D arsenic was predicted via first‐principles calculations. There are many allotropic forms of arsenic. However, the most stable form is gray bulk As with a buckled honeycomb structure.^[^ ^2^, ^18^, ^24^ ^]^ The exotic characteristics of arsenic depend on the number of layers, and they will be changed once the layers are reduced to the 2D limit.^[^ ^25^ ^]^ In general, theoretical results have anticipated that arsenic would display semimetal‐to‐semiconductor transition states and indirect‐to‐direct bandgaps.^[^ ^26^ ^]^ Arsenic in bulk was found to have a bond length, interlayer distance, and bond angle of 0.249 nm, 0.204 nm, and 97.29°, correspondingly.^[^ ^24a^ ^]^ Kamal and co‐workers^[^ ^18b^ ^]^ presented optimized structures of arsenene as shown in **Figure** **8**a–c with appropriate density states for puckered, planar, and buckled arsenene (Figure 8d–f). Wang and co‐workers^[^ ^27^ ^]^ used ab initio many‐body Green's function approach to predict the fundamental band structures and dielectric property of multilayer arsenene as illustrated in Figure 8h,i. They calculated photoluminescence and optical absorption bandgaps of multilayer arsenene from the GW‐BSE approach with a large exciton binding energy of 0.8 and 0.9 eV, respectively.

Fei and co‐workers^[^ ^24a^ ^]^ theoretically analyzed the phonon bands of monolayered arsenene. They confirmed that arsenene has significant kinetic stability. They demonstrated that monolayered arsenene had buckled honeycomb structural chemistry in which every atom of arsenic is joined by three other atoms and presented layer‐on‐layer morphology. The DFT simulations on monolayered arsenene's electrical characteristics revealed that it abruptly changed from semimetal to semiconductor with a bandgap of 2.49 eV. The second‐order effects of the interfacial interaction on layer thickness and the quantum confinement effects are primarily responsible for the underlying mechanism of this transformation. More intriguingly, under relatively negligible strain, the indirect bandgap of monolayered arsenene can change to a direct bandgap which opens the doors for 2D arsenene's extremely effective application in optoelectronics. Wang and co‐workers^[^ ^27^ ^]^ employed ab initio GW‐BSE quantum transport approaches and the DFT electron–phonon interactions approach to investigate the absorption coefficient, bandgap, and charge carrier density of monolayer arsenene.

Figure 8d depicts the monolayer arsenene's hypothetical portion of the dielectric property calculated with and without electron–hole coupling, illustrating the distinction between the intrinsic bandgap and the absorption spectrum bandgap. Moreover, monolayer arsenene‐based FET exhibits outstanding performance, which is predicated on the ab initio quantum transport methodology, demonstrating the future use of monolayer arsenene on nanoscale electronic and optoelectronic devices. Furthermore, Zeraati and co‐workers^[^ ^28^ ^]^ used ab initio simulations to examine the crystalline conductivity of 2D arsenene. They found that the anisotropic heat capacities of arsenene with the puckered structure were 30.4 and 7.8 W m^−1^ K^−1^ with diagonal and armchair patterns (Figure 8e), correspondingly.

### 3.3 Antimonene

**Figure 9:**
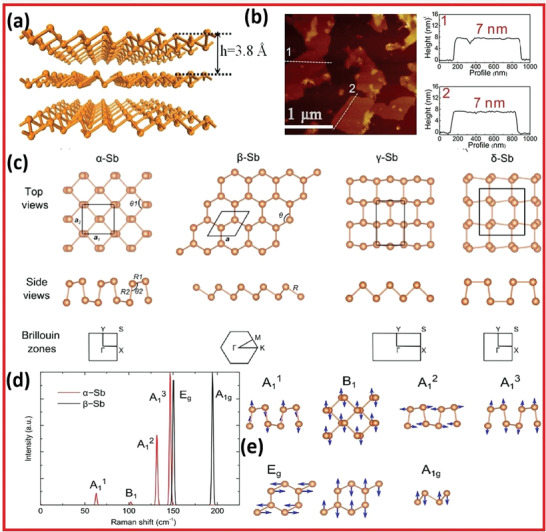
a) Antimonene's structural chemistry. b) AFM image of antimonene. Reproduced with permission.^[^ ^30^ ^]^ Copyright 2018, American Chemical Society. c) Structural configurations of Sb. d) Raman spectra of Sb. e) Allotropes vibrational approaches. Reproduced with permission.^[^ ^1d^ ^]^ Copyright 2015, American Chemical Society.

The indirect nature of the bandgap of antimonene has been demonstrated.^[^ ^27^, ^29^ ^]^ Based on DFT calculations,^[^ ^1d^ ^]^ antimonene has stable free‐standing allotropes. **Figure** **9** depicts puckered antimonene's double sublayers. It has exhibited unusual properties, including high charge carrier mobility, higher constancy, and adjustable bandgap. The multilayered structure is formed through vdWs force, with a layer spacing of 0.38 nm, as shown in Figure 9a.^[^ ^30^ ^]^ AFM images of antimonene are presented in Figure 9b.

In contrast to the TMDs, the change in bulk Sb into monolayer is followed by a shift in electrical characteristics (from semimetallic to semiconducting with an indirect bandgap of 2.28 eV).^[^ ^3a^ ^]^ Surprisingly, the biaxial pressure could convert freestanding antimonene from an indirect bandgap into a direct bandgap. DFT‐based theoretical calculations with or without SOC consideration reported a wide variety of bandgap values for monolayer antimonene from the top viewpoint (the computed bond lengths are 0.283 and 0.291 nm, while the bond angles are 95.0° and 102.5°). The buckled hexagonal lattice of antimony possesses bond lengths of 0.284 nm and bond angles of 89.9°, as illustrated in Figure 9c. Raman spectroscopy is commonly used to characterize 2D materials. Raman spectra of both single‐layered antimonene and bulk Sb have been compared.^[^ ^1d^ ^]^ Raman spectra for *α*‐Sb and *β*‐Sb monolayers are demonstrated in Figure 9d. For *α*‐Sb, the out‐of‐plane modes (A_1_^1^ at 63 cm^−1^ and A_1_^3^ at 147 cm^−1^) and in‐plane modes (B_1_ at 102 cm^−1^ and A_1_^2^ at 132 cm^−1^) display Raman scattering. For the A_1_^1^ mode, atoms in the same sublayer vibrate in opposite directions. A_1_^3^ is the most important Raman peak for *α*‐Sb for which atoms in the same sublayer vibrate in the same direction and the two sublayers vibrate opposite to each other. For *β*‐Sb, the Raman active modes are at 150 cm^−1^ (in‐plane E_g_ mode) and 195 cm^−1^ (out‐of‐plane A_1g_ mode).^[^ ^1d^ ^]^ Lin and co‐workers conducted experimental and theoretical research on Raman spectroscopy for few‐layer antimonene and bulk antimony.^[^ ^31^ ^]^ The Raman spectra of bulk antimony stimulated by a 532 nm laser exhibit two primary vibration peaks at 110 and 149.8 cm^−1^, which correspond to a pair of degenerate in‐plane transversal and longitudinal modes of Eg and out‐of‐plane mode of A_1g_, respectively.^[^ ^25^ ^]^ The relationship between Raman strength and thickness of the flake was examined. It was discovered that peak intensities fell as thickness reduced, and the Raman signal might not be noticed with thickness less than 70 nm. They also tried to investigate the effect of excitation laser wavelength (785, 633, 532, 473, 457, and 405 nm), however, no Raman signal was detected for the flake with a thickness of 10 nm. Furthermore, theoretical phonon spectra simulations projected a significant contraction of the in‐plane lattice constant from antimony (bulk) with *a* = 0.43 nm toward single‐layer antimonene with *a* = 0.401 nm, leading to blue shift values for E_g_ and A_1g_ patterns.

**Figure 10:**
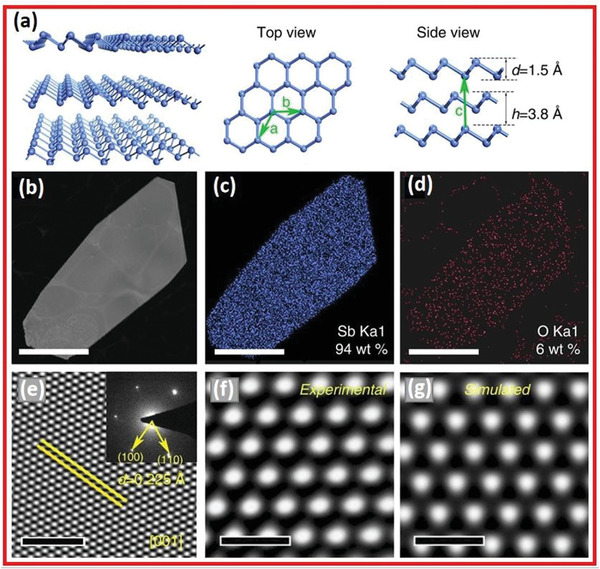
a) Diagrammatic representations of the *β*‐phase antimonene. b) TEM image. c) Sb is calculated to be 94 wt%. d) O is calculated to be 4 wt%. e) SAED pattern. f) HRTEM of multilayered antimonene. g) Simulated multi‐layered antimonene. Reproduced with permission.^[^ ^25^ ^]^ Copyright 2016, Springer Nature.

There are many allotropes of antimony, and one of the most reliable allotropes has a rhombohedral structure (*β*‐phase) resembling that of gray arsenic. **Figure** **10**a demonstrates the antimony crystal structures in the *β*‐phase.^[^ ^25^ ^]^ Antimonene in the single‐layer *β*‐phase is made of buckled hexagonal rings of Sb atoms joined by sp^3^ bonds and exhibits the lowest average binding energies. A single‐layer polygonal antimonene is depicted in Figure 10b with a high‐angle annular dark‐field (HAADF)‐STEM image. According to the energy dispersive spectroscopy (EDS) elemental map in Figure 10c,d, the crystal is primarily made of Sb with a trace amount of oxygen. As the value is much lower than that in antimony oxide, the oxygen content of approximately 6 weight percent is attributed to groundwater pollution during the transfer process. By contrasting selected area electron diffraction (SAED) patterns, it's evident that the *β*‐phase could be differentiated from the *α*‐phase. Figure 10e purely affirms the framework of b‐phase observing all along [001] area axial direction, implying that the 2D Sb crystal is rhombohedral (*β*‐phase) instead of orthorhombic (*α*‐phase). The high‐resolution transmission electron microscope (HRTEM) image can be seen in Figure 10e, and an amplified image is shown in Figure 10f. The simulated and experimental results agree so well on the configuration of bright points, verifying the ABC stacking of the antimonene layers. The above findings demonstrate that obtained antimonene polygons have the expected rhombohedral structure (*β*‐phase).

### 3.4 Bismuthene

Nagao and co‐workers^[^ ^32^ ^]^ successfully grew the monoelemental bismuthene on a silicon wafer in 2005. Bismuthene has shown interesting semiconductor properties and received a great deal of attention in recent decades due to its beneficial properties.^[^ ^33^ ^]^

**Figure 11:**
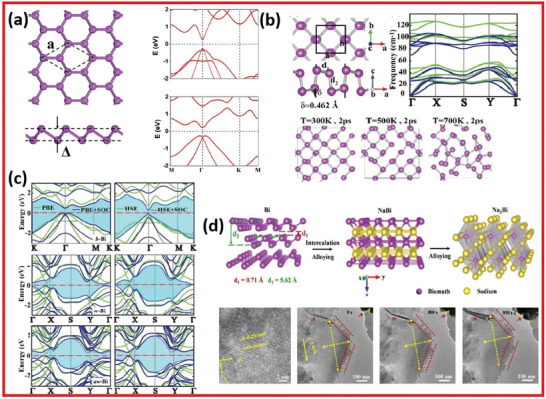
a) Bismuthene and its band structures calculated with and without SOC. Reproduced with permission.^[^ ^34^ ^]^ Copyright 2017, American Chemical Society. b) Bismuthine band structures with energy diagram. c) Free‐standing energy band assemblies. Reproduced with permission.^[^ ^35^ ^]^ Copyright 2016, American Chemical Society. d) In situ TEM images of Na insertion in bismuthene during electrochemical sedation in Na‐ion batteries. Reproduced with permission.^[^ ^113^ ^]^ Copyright 2019, Wiley‐VCH.

The structure of bismuthine has been investigated,^[^ ^34^ ^]^ and it is justified that bismuthine has buckled 2D hexagonal structure through a constant lattice value of *a*  = 4.36 Å. The bonding length between Bi—Bi is 3.05 Å, and the buckled bismuthene in various views is illustrated in **Figure** **11**a. The honey‐comb buckled bismuthine shows excellent stability. Aktürk and co‐workers^[^ ^35^ ^]^ presented the first principle calculations for bismuthene structure (Figure 11b). Moreover, they calculated the energy band structures of bismuthine (*β*‐Bi, *w*‐Bi, and *aw*‐Bi) as shown in Figure 11c. The *β*‐phase of the crystalline bismuth (in rhombohedral layered structure) is known as metallic bismuthene. Normally, the rhombohedral phase of bismuth is stable under air conditions.^[^ ^2d^ ^]^ Due to the quantum confinement effect, semimetal bismuth is transformed into a semiconductor as its dimensions are scaled down to nanostructures, which is very attractive for optoelectronic devices.^[^ ^36^ ^]^ Various methods have been employed to create bismuth nanostructures such as nanosheets, nanowires, nanorods, nanotubes, and nanospheres.^[^ ^37^ ^]^ Kumar and co‐workers^[^ ^38^ ^]^ prepared 2D bismuthene nanosheets via wet chemical reduction at low temperature in 2013. Gusmão and co‐workers^[^ ^39^ ^]^ adopted the aqueous shear exfoliation technique to prepare 2D bismuthene nanosheets. Lu and co‐workers^[^ ^40^ ^]^ developed a simple process to prepare few‐layered bismuthene by sonochemical exfoliation. Furthermore, Yang and co‐workers^[^ ^41^ ^]^ created an innovative technique that combines ultrasonication and acid interaction for exfoliating layered semiconducting bismuthene. First, they mixed specific quantity bismuth (bulk) and (NH_4_)_2_S_2_O_8_, followed by the sequential addition of H_2_SO_4_ and H_2_O_2_. Then the above‐mentioned dispersed material was maintained for 6 h and subsequently washed away by anhydrous ethanol to take off H_2_SO_4_. Following vacuum drying, the powder was dispersed in N‐methyl‐2‐pyrrolidone (NMP) solution in an inert environment. Once the powder turned brown, the nanosheets were extensively collected by centrifugation. The strong interaction of H_2_SO_4_ in this strategic approach causes bismuth to develop and exfoliate into relatively small nanosheets.

Most notably, Reis and co‐workers^[^ ^42^ ^]^ have successfully synthesized single‐layer bismuthene on a SiC(0001) substrate in honeycomb geometry, as predicted theoretically.^[^ ^43^ ^]^ Combined with the theoretical investigation based on DFT including the strong SOC, the large gap quantum spin Hall (QSH) phase in bismuthene is confirmed and stabilized by the effect of substrate, highlighting the important role of substrate coupling for the epitaxy of 2D bismuthene.^[^ ^44^ ^]^

## 4 Synthetic Approaches and Characteristics

The atomically thin layers of 2D materials with superior electrical, optical and mechanical characteristics have tremendous potential applications. Credible 2D material synthesis is essential for real‐world applications in microelectronics and optoelectronics. There are many synthesis techniques for 2D group VA semiconductors. They are frequently categorized into two parts: top‐down and bottom‐up. Exfoliation (mechanical, chemical, and liquid‐phase exfoliation) is an example of a top‐down technique. Chemical vapor deposition (CVD), physical vapor deposition (PVD), wet chemical methods, and pulsed laser deposition (PLD) are all included in the bottom‐up approaches for creating 2D materials.

Three processes are frequently used to develop 2D materials: mechanical cleavage, PVD, and CVD. Exfoliating single‐ and few‐layer sheets from the mass crystal can be done using micromechanical cleavage, also referred to as the Scotch tape approach. This method yields high‐quality crystalline samples suitable for electrical and optical devices. Recent advancements in exfoliation methods can extract, shape, and stack sheets with controlled thickness and alignment.^[^ ^45^ ^]^ The PVD procedure deposits material on a substrate by using atomic sources. Ultrahigh vacuum (UHV) and cleansed atomic source materials are usually needed for PVD. In addition, specimens made under UHV situations could be investigated in situ using surface‐sensitive techniques.^[^ ^46^ ^]^ CVD utilizes the reaction of gases, liquids, or solid species in a controlled atmosphere.^[^ ^47^ ^]^ Typically, nucleophilic substances are the right choice for CVD, and the substrate has an impact on the growth procedure.

### 4.1 Top‐Down Approach

The top‐down approaches include mechanical exfoliation, ultrasonic exfoliation, plasma etching hot‐pressing, electrochemical treatment, etc. A physical or/and chemical method is used in top‐down synthesis to create ultrathin nanolayers. The top‐down physical technique exfoliates layered 2D materials bulk into single or few‐layer by applying the mechanical power of ultrasonic vibrational forces. The chemical method is largely concerned with chemical reactions produced by electrostatic contact, heating, and other mechanisms.

#### 4.1.1 Mechanical Exfoliation

**Figure 12:**
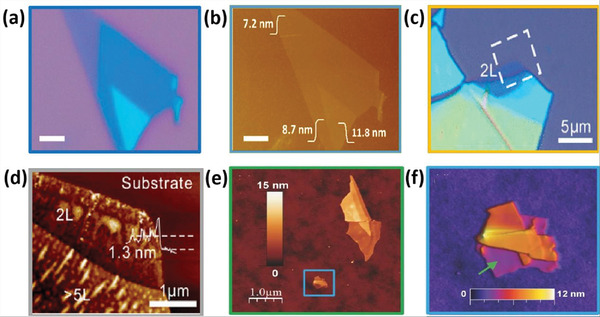
a) Optical image of a BP nanosheet. b) AFM images of a BP nanosheet. Reproduced with permission.^[^ ^49^ ^]^ Copyright 2015, American Chemical Society. c) Optical image of exfoliated phosphorene. d) AFM image of phosphorene. e) Topographic antimonene image. Reproduced with permission.^[^ ^50^ ^]^ Copyright 2014, American Chemical Society. f) AFM image of the antimonene. Reproduced with permission.^[^ ^51^ ^]^ Copyright 2016, Wiley‐VCH.

Mono‐ to few‐layer BP was exfoliated and applied in transistors in 2014 using a conventional exfoliation process.^[^ ^48^ ^]^ Miao and co‐workers^[^ ^49^ ^]^ used a mechanical exfoliation process to fabricate a BP‐based FET device. **Figure** **12**a presents the optical images of BP flakes, and Figure 12b shows an atomic force microscopy (AFM) image of the same BP nanoflake with a scale bar of 2.5 µm. Zhang and co‐workers^[^ ^50^ ^]^ fabricated few‐layer phosphorene flakes via the mechanical exfoliation technique. The flakes were first distinguished in a microscope using optical contrast. Colored regions refer to different thicknesses of phosphorene flakes. A representative two‐layered thin phosphorene on a Si/SiO_2_ substrate is shown in Figure 12c (using an optical microscope). The number of layers could be determined by AFM (Figure 12d). Ares and co‐workers^[^ ^51^ ^]^ used freshly sliced crystals of antimony for single to few‐layer flake preparation by mechanical exfoliation approach. They used adhesive tape and pealed the sub‐millimeter flakes of antimony by repetitive exfoliation. These flakes can be transferred on a silicon wafer with 300 nm oxide by putting the scotch tape in the opposite direction to the substrate. Then, to make handling easier, a further approach was introduced. The flakes could be transferred from sticky tape to a thin layer of viscoelastic polymer which was adhered to the glass slide. By pressing the polymer against the substrate with the help of optical microscopy, researchers can acquire thin antimony flakes in a controlled manner. The 2D group VA members with rhombohedral structures (As, Sb, Bi, and P) are brittle, and they will be rapidly pulverized.^[^ ^52^ ^]^ The Scotch tape approach was applied for *β*‐arsenene (rhombohedral) with high anisotropy. Mechanical exfoliation was used to remove gray Sb layers.^[^ ^51^ ^]^ In addition, single‐ and few‐layered *β*‐arsenene could be synthesized via liquid‐phase exfoliation (LPE) and bottom‐up approaches.^[^ ^53^ ^]^

**Figure 13:**
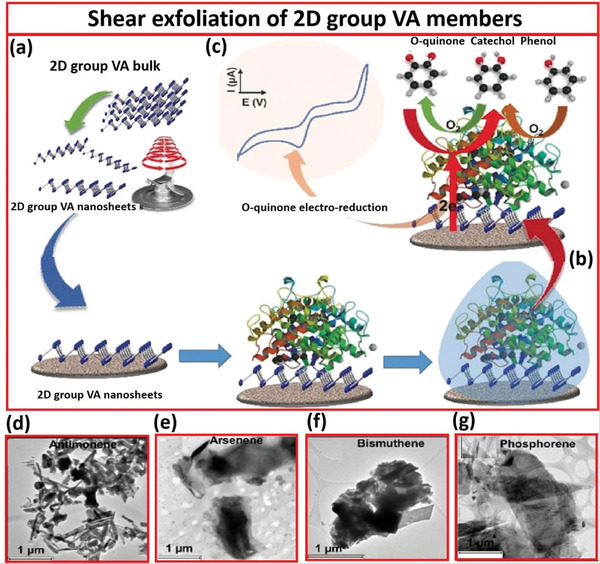
a) Shear exfoliation of arsenene, bismuthine, and phosphorene via kitchen blender. b) Fabrication of biosensor via layer‐by‐layer 2D VA group members. c) Chemical mechanism of phenol detection by a biosensor based on exfoliated 2D group VA members and Tyr. d) TEM image of antimonene. e) TEM image of arsenene. f) TEM image of bismuthene. g) TEM image of phosphorene. Reproduced with permission.^[^ ^54^ ^]^ Copyright 2020, Wiley‐VCH.

Mayorga‐Martinez and co‐workers^[^ ^54^ ^]^ produced enzyme‐mediated sensors based on the bulk and exfoliated 2D group VA materials (phosphorene, antimonene, arsenene, and bismuthene), and investigated the biosensing properties.^[^ ^54^ ^]^ **Figure** **13** depicts the development and implementation of these biosensors. A kitchen blender was used for the shear exfoliation of 2D materials. The nanosheets were then dropped onto a glassy carbon electrode and added with the biochemical modifiers Tyr and Glu. The biosensors can be used to detect phenols electrochemically. Compared to the other representatives of the 2D group VA semiconductors, the exfoliated antimonene‐based biosensor demonstrated the highest levels of linear regression, responsiveness, specificity, and replicability.

**Figure 14:**
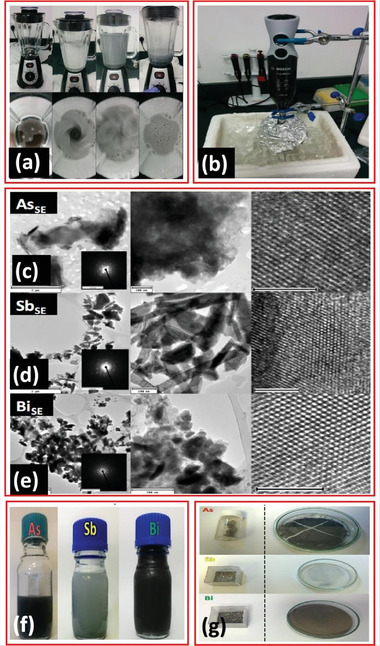
a,b) Photographs of the workflow for exfoliation of 2D group VA materials by shear force exfoliation in aqueous surfactant. c–e) TEM and HRTEM images of the different bulk and shear exfoliated materials. f,g) Optical image of the samples. Reproduced with permission.^[^ ^55^ ^]^ Copyright 2017, Wiley‐VCH.

The spinning blade blenders in **Figure** **14**a,b can be used to shear and exfoliate bulk material into single‐ to few‐layer materials inside the liquid phase. Gusmão and co‐workers^[^ ^55^ ^]^ fabricated single‐ to few‐layered arsenene, antimonene, and bismuthene utilizing the aqueous shear exfoliation technique. The experimental approach for arsenene nanolayers is introduced here, identical to that for antimonene and bismuthene. Before purifying the granules, the materials need to be processed to remove the surface oxides. For twenty minutes, 1 g of As powder is sonicated inside an ice bucket using 5 mg mL^−1^ of sodium cholate as the aqueous surfactant. The residue is then evaporated at 60 °C after centrifugation for 1 h at 4000 rpm. The pre‐treated arsenic particles are combined with sodium cholate surfactant during the shear dispersing and exfoliation approach. As indicated in the figure, the exfoliation cycle continues for 2 h, which is switching between two kitchen mixers. The TEM and HRTEM images of the bulk and shear exfoliated materials are shown in Figure 14c,d.

Jacobs‐Gedrim and co‐workers^[^ ^56^ ^]^ reported the 2D Sb_2_Te_3_ nanosheets from 6 mm lumps, and these lumps were cleaned with a sharp blade to expose the clear outer surface. The Sb_2_Te_3_ nanosheets were transferred onto a substrate with fixed interdigitated electrodes after micromechanical exfoliation. Teweldebrhan and co‐workers^[^ ^57^ ^]^ investigated Bi_2_Te_3_ as well, and the layered counterparts were got by mechanical cleavage exfoliation. Mechanical exfoliation was also used to exfoliate black arsenic‐phosphorus (*β*‐AsP) layered materials to atomic layers.^[^ ^75^ ^]^ A vapor transport method was used to prepare bulk samples, which were then exfoliated into flakes using Scotch tape.

#### 4.1.2 Ultrasonic Exfoliation

**Figure 15:**
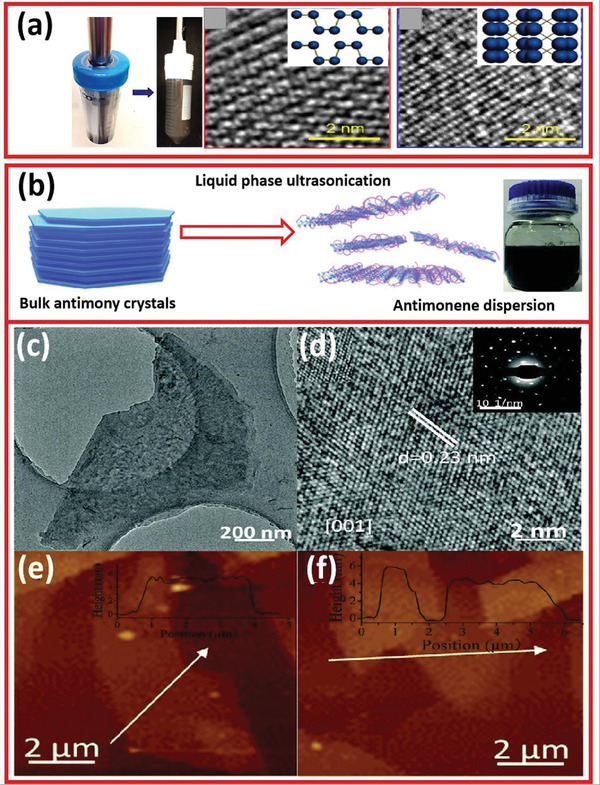
a) Optical image of the samples and HRTEM image of phosphorene after exfoliation. b) Liquid‐phase exfoliation of bulky antimony into antimonene. c) TEM images of antimonene. d) HRTEM with SAED pattern. e,f) AFM images. Reproduced with permission.^[^ ^59b^ ^]^ Copyright 2015, American Chemical Society.

Ultrasonic‐assisted liquid phase stripping is a viable technique. Dimethyl formamide (DMF), isopropyl alcohol (IPA), N‐cyclohexyl‐2‐pyrrolidone (CHP), dimethylsulfoxide (DMSO), NMP, etc. are commonly used to disperse bulk layered crystals.^[^ ^58^ ^]^ A pyrolysis reaction of hydroxyl radical mediating ultrasonic cavitation leads to numerous bubbles on the crystal surface, resulting in the formation of ultrathin 2D nanomaterials. The nanosheets with high purity and uniform size were prepared using an ultrasonic probe via LPE in both NMP and CHP under cooling conditions (**Figure** **15**a).^[^ ^59^ ^]^ HRTEM image of phosphorene is also presented in Figure 15a. The preparation approach, TEM image, HRTEM image with SAED pattern, and AFM image of antimonene are illustrated in Figure 15b–f.^[^ ^59b^ ^]^ By varying the LPE power and reaction time, phosphorene quantum dots (QDs) were prepared by Song and co‐workers.^[^ ^60^ ^]^ The phosphorene QDs have an average size and thickness of about 3.5 to 4.5 nm and 1.2 to 1.6 nm. The LPE method can also prepare 2D arsenene in NMP solvent from gray arsenic. The as‐prepared material has approximately 6 to 12 layers, as confirmed by Raman spectra analysis.^[^ ^61^ ^]^ With IPA and water at 400 W, the transverse size of *β*‐antimonene can be greater than 1–3 µm^2^ with a thickness of 4 nm.^[^ ^62^ ^]^ Tao and co‐workers developed QDs using an ice‐bath shaker and probe sonication in a two‐step process. The QDs were modified with 1,2‐Distearoyl‐sn‐glycerol‐3‐phosphoethanolamine‐N‐[methoxy (polyethylene glycol)] (DSPE‐mPEG) to increase the dispersion speed and stability. It has a thickness of about 1.6 nm with an average size of about 2.8 nm.^[^ ^63^ ^]^ This method also could synthesize ultrathin bismuthene QDs using the NPM solvent at 400 W.^[^ ^64^ ^]^ 2D selenium can also be prepared with a thickness of <10 nm and dimensions of 20 to 130 nm using 200 W using the LPE approach.^[^ ^65^ ^]^ Similarly, 2D tellurium can be synthesized in an IPA medium after pulverizing by LPE. The stripping process can highly maintain the crystallization properties of tellurium nanosheets.^[^ ^66^ ^]^ The ultrasonic speed and reaction time are key factors in controlling the size and thickness of 2D materials. However, the role of the solvent medium cannot be overlooked. It is normally embedded into the interlayer, breaking van der Waals forces to obtain nanomaterials. Thus, the ultrasonic and reaction parameters are critical in controlling the size and thickness of 2D group VA materials.^[^ ^67^ ^]^

**Figure 16:**
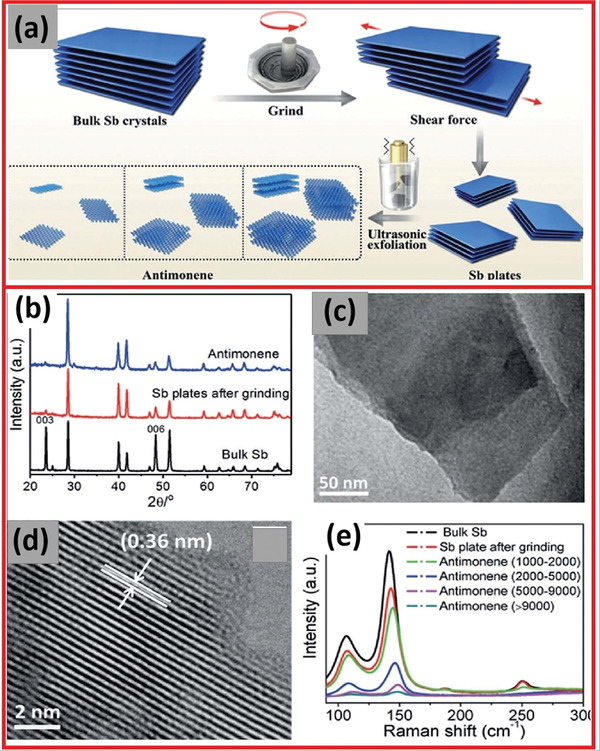
a) The synthesis approach of antimonene (Sb bulk to monoelemental layer). b) XRD spectra of antimonene. c) TEM and d) HRTEM images of antimonene. e) Raman spectra (bulk Sb and monolayered antimonene). Reproduced with permission.^[^ ^68^ ^]^ Copyright 2017, Wiley‐VCH.

Wang and co‐workers^[^ ^68^ ^]^ described sample pre‐grinding and ultrasonication‐assisted LPE for the preparation of antimonene. In the presence of 2‐butanol, the pre‐grinding process produces large and thin Sb plates, typically exfoliated into large and smooth antimonene sheets, shortening the sonication time (**Figure** **16**a). XRD peak which was attributed to the 003 facets confirmed that the bulk Sb crystals were successfully exfoliated. The decrease in XRD peaks suggested that antimonene was produced through exfoliation along the *c*‐axis (Figure 16b). The antimonene has a smooth structure, and its surface is primarily covered by facets (Figure 16c,d).^[^ ^69^ ^]^ The interplanar distance between its lattice fringes was 0.36 nm.^[^ ^103^ ^]^

In contrast, the LPE‐created antimony showed a broader layer distribution, which yielded various layered structures, including a remarkable Tyndall phenomenon. The higher speed of ultrasonication could form thinner size antimonene with a much lower yield and good stability. The Raman spectra revealed the two bands caused by the bulk Sb crystal's E_g_ and A_1g_ vibration modes (Figure 16e).

**Figure 17:**
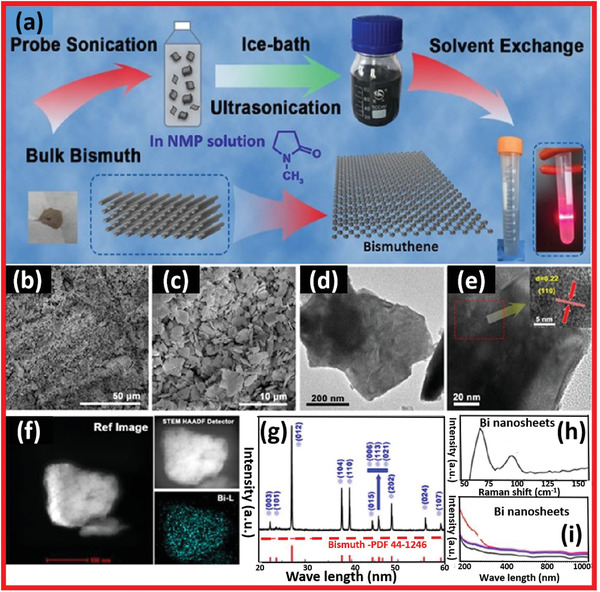
a) Few‐layered bismuthine syntheses via hydrothermal approach. b,c) Low and high magnified SEM images. d,e) TEM image of few‐layered bismuthene. f) EDS mapping. g) XRD spectra. h) Raman spectrum. i) UV–vis spectra of few‐layered bismuthene. Reproduced with permission.^[^ ^33a^ ^]^ Copyright 2018, Institute of Physics.

**Figure 18:**
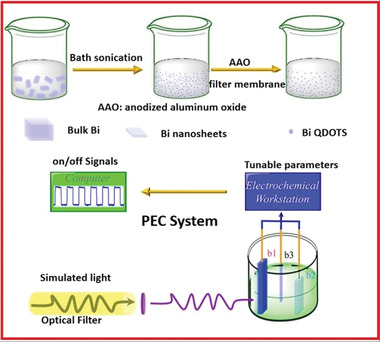
Bi quantum dots based photodetectors: Synthesis via liquid‐phase exfoliation and PEC system constructed for assessing the device with photo response. Reproduced with permission.^[^ ^64^ ^]^ Copyright 2015, American Chemical Society.

Bi powder was also used in the ultrasonication process to create Bi nanosheets (**Figure** **17**a). Bi powder was added to 300 mL NMP solvent, which was then ultrasonicated using an ice‐bath sonicator at 600 W for the first 6 h and then 24 h at 188 W.^[^ ^33a^ ^]^ When exposed to air, Bi was oxidized. Because of its excellent oxidation properties, NMP was used as an organic solvent for exfoliation. The exfoliated sheets had uniformly distributed dimensions (Figure 17b,c) and an interconnected lamellar structure (Figure 17d,e). A lattice spacing of 0.22 nm represents the lattice plane of 110. The EDS mapping results show that the Bi is in a specific location within the structure (Figure 17f). XRD results show that hexagonal Bi nanosheets are highly pure (Figure 17g).^139^ The Raman spectra confirm that the peak positions at 65.6 and 92 cm^−1^ correspond to the Eg and A1g mode vibrations, which are consistent with Bi nanosheet properties (Figure 17h).^138^ This is also confirmed by atomic absorption spectra (Figure 17i). The Bi nanosheet thickness was calculated to be 6 to 12 nm. Xing and co‐workers^[^ ^64^ ^]^ adopted LPE for Bi QDs fabrication, as presented in **Figure** **18**. The bulk Bi powder was subjected to bath sonication in a solution of NMP (5 mg mL^−1^). By using built‐in cooling water machinery, the temperature was kept at 5 °C. The combination mentioned above was first sieved via a permeable anodized aluminum oxide membrane (100 nm pore diameter) to eliminate large‐size Bi nanoparticles and then sieved through 20 nm pore diameter membrane to yield mono‐layered Bi nanosheets. The Bi nanosheets in NMP solution were centrifugated for 30 minutes at 18 000 rpm. In a vacuum‐drying oven, the Bi QDs‐based residue was dried. 1 mg of Bi‐based nanosheets were disseminated in 1 mL of poly(vinylidene fluoride) (PVDF)/NMP solution by ultrasonication at 400 W for 30 min as presented in Figure 18. The mixture was placed directly on the indium tin oxide (ITO) glass and then annealed inside a vacuum oven at 80 °C for 24 h.

#### 4.1.3 Plasma Etching

Etching is a method for eliminating foreign material by physical or chemical methods. Chemical wet etching, plasma dry etching, and various physical and chemical etching methods are examples of etching approaches.^[^ ^1^, ^70^ ^]^ Due to the obvious unique plasma‐material interactions that could facilitate the greater transfer of energy at the nanoscale, plasma is indeed an effective approach to synthesizing and modifying 2D materials. In plasma etching, a sample is blasted at a very high speed with a glow discharge of the proper gas composition. The plasma source could be either chargeable or neutral. Moreover, it has shown that the fabrication of functional devices requires the application of etching approaches to manage the layer number and organize the shape of layered 2D materials.^[^ ^70^, ^71^ ^]^ In the 1960s, the plasma etching approach was used to manufacture electronic components. At the moment, large‐scale integrated circuit production uses low‐temperature plasma extensively.^[^ ^72^ ^]^

**Figure 19:**
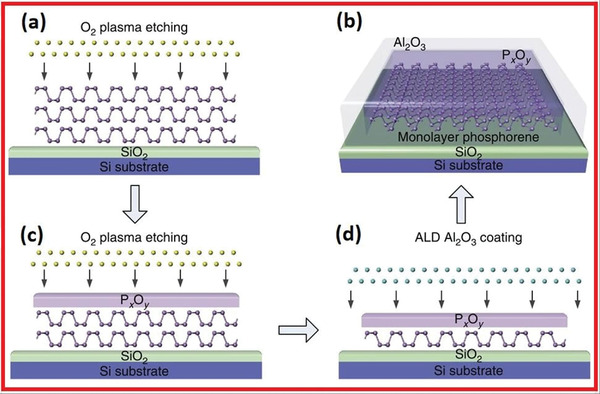
a) Phosphorene by oxygen plasma etching. b) The upper single‐layers of the phosphorene nanoflakes are being oxidized (P*_x_* O*_y_*). c) Due to the consistent etching frequency, sufficient numbers of phosphorene layer upon layer down to a monolayer could be accurately fabricated. d) ALD has been used to encase the specimen with just an Al_2_O_3_ protective layer. Reproduced with permission.^[^ ^73^ ^]^ Copyright 2016, Springer Nature.

Pie and co‐workers^[^ ^73^ ^]^ presented the etching method for synthesizing air‐stable phosphorene. **Figure** **19**a depicts the very thin layers of phosphorene via high oxygen plasma etching. The next step is the subsequent passivation procedure. Few‐layered phosphorene is placed on the upper side of the SiO_2_/Si substrate. The outer layers of the phosphorene crystals have been oxidized to P*_x_* O*_y_* during the O_2_ plasma pre‐treatment process. This represents a protective coating for residual phosphorene specimens. O_2_ plasma etching pierces the P*_x_* O*_y_* layer and oxidizes the underpinning phosphorene, which also thins the phosphorene layer while increasing the P*_x_* O*_y_* thickness. In the meantime, the “high oxygen plasma” physically fizzles aside the P*_x_* O*_y_* thin coating from the top due to oxygen plasma crashes. Following the plasma pretreatment, a dynamic equilibrium between phosphorene oxidation and physical removal of the P*_x_* O*_y_* layer is reached. Therefore, the P*_x_* O*_y_* layer approaches a steady thickness, and the etching rate reaches constant, as shown in Figure 19b. Due to the obvious continual etching frequency, a sufficient number of phosphorene layers down to a monolayer can then be manufactured (Figure 19c). The deterioration is hindered due to the controlling nature of the P*_x_* O*_y_*. The durability of phosphorene has been enhanced. The specimen was encased with an Al_2_O_3_ protective coating via atomic layer deposition (ALD). In this particular process, the P*_x_* O*_y_* coating protects the underpinning phosphorene from ALD precursor gases, which are particularly important for specimens that are only a few‐layer thick (Figure 18d).

#### 4.1.4 Hot‐Pressing Method

The 2D nanomaterials of semimetals, metalloids, polymeric materials, and oxides of different metals are difficult to fabricate because of their inherent property without layer lattice fringe. Wet chemical techniques and molecular beam epitaxy (MBE) are the most common ways of producing 2D layered materials. Low availability, severe circumstances, unique substrate conditions, and changes in fundamental material characteristics due to interface interaction with the substrate may limit the application of the MBE growth approach.^[^ ^74^ ^]^ Some complex stages are involved in wet chemical procedures, which make nanosheets in minor parts. In addition, surfactants are inappropriate for electronic devices, spintronics, and catalysis applications. Investigation of substitute ways for obtaining huge surface area, good quality, large and ultrathin nanostructured material is required, as it aids research into important characteristics and intriguing applications. Hot‐pressing large‐area and ultrathin nanosheets from extremely crystalline material is a modest and cost‐effective mechanical approach.

**Figure 20:**
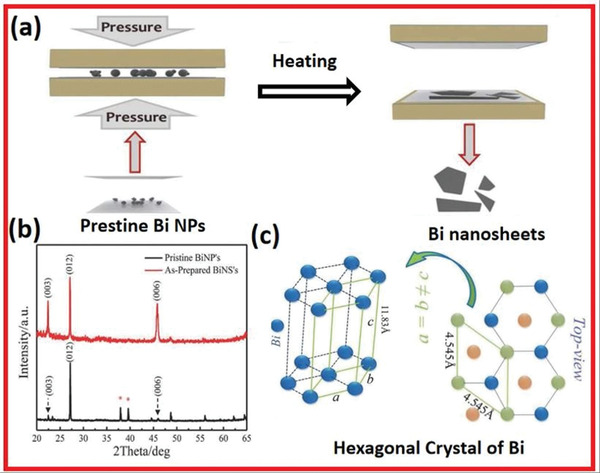
a) Hot‐pressing approach showing Bi nanosheet synthesis. b) XRD spectra of Bi bulk and Bi nanosheets. c) Hexagonal crystal structure with a rhombohedral A7 unit cell and its lattice gap. Reproduced with permission.^[^ ^75^ ^]^ Copyright 2017, Wiley‐VCH.

Hussain and co‐workers planned a low‐cost mechanical method for fabricating ultrathin bismuthine nanoflakes through crystalline Bi (**Figure** **20**a).^[^ ^75^ ^]^ Utilizing a simple hot‐pressing approach, ultrathin monolayers of Bi can be fabricated on Si substrates. The Si substrate was first dropped with Bi nanoparticles dispersed in ethanol solution. The Bi nanoparticles were placed between two Silicon substrates after drying. The pair was then sandwiched between steel plates from a hot‐press machine. The pressure was raised from ambient to 0.54 GPa, while the plate's temperature was raised from room temperature to 150 °C. At a constant pressure of 0.54 GPa, the Silicon substrates were pressed at 150 °C for 30 min before being naturally cooled to room temperature. A low operating temperature was chosen to prohibit oxide formation. In any instance, no postfabrication action was necessary. Bi nanosheets were successfully produced by compressing small agglomerates of Bi nanoparticles at suitable temperature and pressure settings.

**Figure 21:**
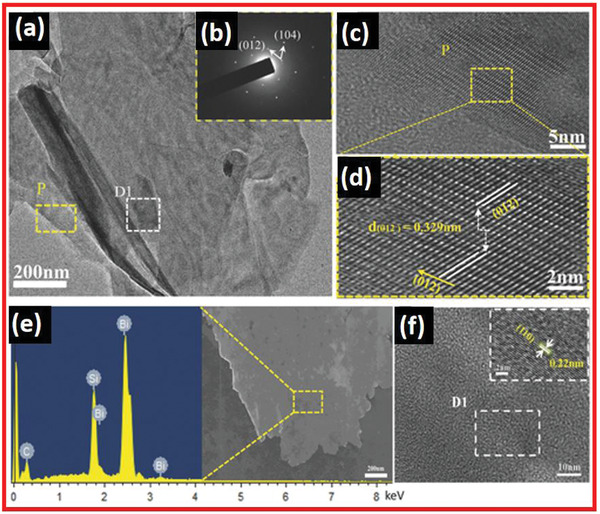
a) TEM image of bismuthine. b) SAED pattern. c) HRTEM image of bismuthene. d) Crystal structure construction of Bi (012) and suitable lattice fringes in HRTEM image. e) EDS mapping. (f) HRTEM image. Reproduced with permission.^[^ ^75^ ^]^ Copyright 2016, Wiley‐VCH.

The hexagonal crystalloid indexed XRD peaks of Bi are shown in Figure 20b. The result agreed well with conventional literature values from JCPDS Card no. 05‐0519 (Figure 20c). There was correspondingly no peak associated with the oxidation stage of Bi. When compared to the XRD patterns of Bi nanoparticles, the (003) and (006) peak intensities of the Bi nanosheet samples were increased. The texture of the materials is extremely crystalline and can withstand a great deal of plastic deformation and undergo microstructure alterations. The alignments of single crystals vary during deformation with the direction of applied force. Stress creates the recrystallization of Bi nanosheet along a favored 001 plane, as shown by the prominent reflections in diffraction peaks (003 and 006). The main crystal phase is the 012 plane, which carries bulk characteristics into the nanosheets. The XRD results indicated that the Bi nanosheets were well textured and highly orientated primarily along with the 012 planes, which agreed well with the HRTEM (**Figure** **21**a,f) and SAED pattern in Figure 21b. The prepared ultrathin texture Bi nanosheets were semi‐transparent with approximately 2.55 nm diameter and self‐supporting. They confirmed the fabrication of Bi nanosheets on the Silicon substrate through EDS, as presented in Figure 21e. The Bi possesses sheet‐based nanostructured phases, as shown in Figure 21a. The SAED patterns indicate a high degree of crystallinity. The HRTEM image of the lapping D1 part of Bi nanosheets in 110 crystal alignment with 0.22 nm lattice fringe spacing is shown in Figure 21f.

#### 4.1.5 Electrochemical Method

In the synthesis of phosphorene, electrochemical exfoliation has been used.^[^ ^76^ ^]^ When we associate the electrochemical approach with other 2D materials synthetic technologies, this approach may be less expensive for producing 2D materials in industrial production. Furthermore, we can save the need for many chemicals by utilizing the electrochemical approach.^[^ ^77^ ^]^

**Figure 22:**
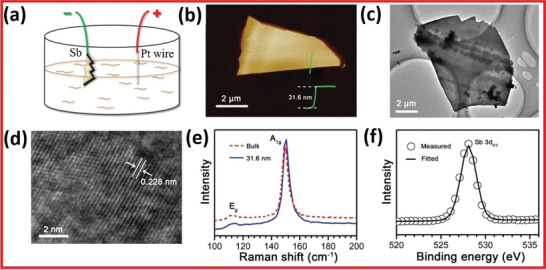
a) Schematic presentation of electrochemical exfoliation of bulk antimony to monoelemental antimonene. b) AFM image presenting antimonene. c) TEM image of antimonene. d) HRTEM image of antimonene. e) Raman spectra of antimonene. f) XPS spectra of antimonene. Reproduced with permission.^[^ ^78^ ^]^ Copyright 2017, Wiley‐VCH.

Lu and co‐workers^[^ ^78^ ^]^ synthesized mono‐ to few‐layered antimony via electrochemical exfoliation.^[^ ^78^ ^]^ They used bulk antimony which was directly linked with the DC power source via conducting wire (**Figure** **22**a). In contrast, the Pt wire was used as the counter electrode. They utilized the aqueous salt of Na_2_SO_4_ as an electrolyte. The working and counter electrodes were moderately dipped in the electrolyte to protect from contamination and electrolysis. The entire procedure was conducted for 60 minutes to achieve thorough intercalation and exfoliation. The electrolyte‐containing exfoliated Sb nanosheets are then centrifuged for 30 min at 6000 rpm. The AFM was used to characterize the freshly prepared Sb sheets (height and phase). The Sb nanoflakes have a smooth surface and an uneven contour with a thickness of 31.6 nm, as shown in Figure 22b.

The lateral scale of the flake in Figure 22c is ≈10.3 µm, and the HRTEM image in Figure 22d also shows a rigid arrangement of lattice planes. The Sb nanosheets show a clear lattice fringe with 0.228 nm interdistance, consistent with rhombohedral gray antimony.^[^ ^25^ ^]^ The bulk antimony (red dashed line) in Figure 22e shows the typical acute scattering peaks of the A_1_ and E_g_ vibration modes located at 149.7 and 111.7 cm^−1^, respectively. When the antimonene is thinned to 31.6 nm, the peak positions of Eg and A1 (the blue lines) are blue‐shifted to 113.8 and 150.9 cm^−1^, respectively. The intensity ratio of the E_g_ and A_1_ peaks drops from 0.07 to 0. 068. The Sb–Sb 3d_5/2_ orbital bonding is confirmed by X‐ray photoelectron spectroscopy (XPS), which exhibits a clear symmetric photoelectron peak at 528 eV (Figure 22f). Since there is just one peak, few‐layer antimonene has a non‐oxidation property.^[^ ^78^ ^]^

### 4.2 Bottom‐Up Approach

The bottom‐up approach includes physical preparation and chemical preparation approaches. Chemical preparation comprises CVD, solvothermal method, whereas physical preparation includes van der Waals and molecular beam epitaxy.

#### 4.2.1 Chemical Vapor Deposition

**Figure 23:**
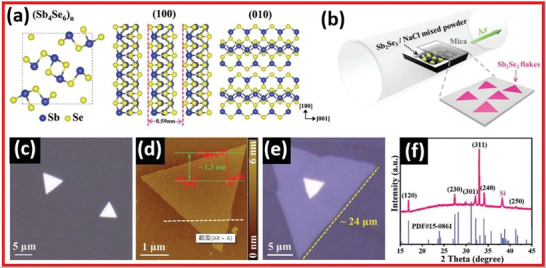
a) Sb_2_Se_3_ (stick‐and‐ball crystal structure) model with various angles. b) Sb_2_Se_3_ on mica. c) SEM image of Sb_2_Se_3_. d) AFM image of Sb_2_Se_3_. e) Ultrathin Sb_2_Se_3_. f) XRD spectra of Sb_2_Se_3_. Reproduced with permission.^[^ ^80^ ^]^ Copyright 2015, Wiley‐VCH.

The CVD approach is a highly effective method for creating first‐class thin films and coatings. Although its application on an industrial scale is widespread, it is always being improved as new materials are introduced. CVD synthesis is being pushed to new heights today and is considered a crucial method for manufacturing homogeneous 2D inorganic materials.^[^ ^79^ ^]^ Zhao and co‐workers^[^ ^80^ ^]^ successfully synthesized 2D ultra‐thin Sb_2_Se_3_ with 1.3 nm thickness via the sodium‐mediated CVD method. The preferred expansion of the (010) plane by the lowermost formation energy is largely responsible for 2D planar geometry. **Figure** **23**a presented Sb_2_Se_3_ (stick‐and‐ball crystal structure) model with various angles. The band structure of 2D Sb_2_Se_3_ nanoflakes reveals a broad absorption band from UV to NIR (300 to 1000 nm), implying that it could be used in broadband photodetection devices. The photodetector based on Sb_2_Se_3_ nanoflakes exhibited exceptional performance, including a broad response ranging from UV to NIR area, demonstrating interesting prospective applications in various fields such as optoelectronics. The SEM image and AFM image of Sb_2_Se_3_ are presented in Figure 23c,d. The ultrathin Sb_2_Se_3_ and XRD pattern of Sb_2_Se_3_ are shown in Figure 23e,f.^[^ ^80^ ^]^

CVD technique has been used to create many 2D groups of VA materials. Phosphorene with an average area of more than 3 µm^2^ can be effectively built from red phosphorus using the CVD technique.^[^ ^81^ ^]^ The film was made up of four layers of phosphorus atoms. Sb_2_Te_3_ nanosheets were also developed in a controllable environment.^[^ ^82^ ^]^ Moreover, the aerogel‐assisted CVD technique could produce monoelemental bismuth oxide films on an amorphous substrate.^[^ ^83^ ^]^ Chang and co‐workers^[^ ^84^ ^]^ created continuous InSe films with a high oxidation resistance environment by using the CVD technique. The processing parameters, for example, the gas pressure in the environment, the gas velocity, the increased rate of temperature, and significant heat protection time, could be altered to control the penetration depth. Since CVD technology is straightforward and reasonably affordable, it is appropriate for massive production and various applications.^[^ ^85^ ^]^

#### 4.2.2 Van der Waals and Molecular Beam Epitaxy

**Figure 24:**
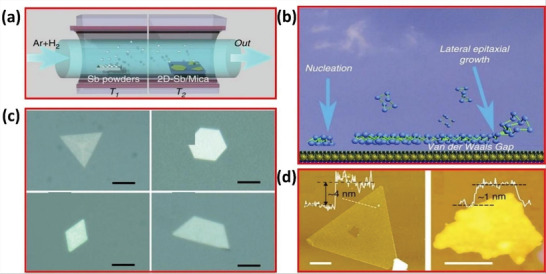
a) The synthesis of antimonene polygons on mica surface. b) Antimonene synthesis via van der Waals epitaxy. c) Optical images of antimonene. d) AFM images of antimonene. Reproduced with permission.^[^ ^25^ ^]^ Copyright 2016, Springer Nature.

**Figure 25:**
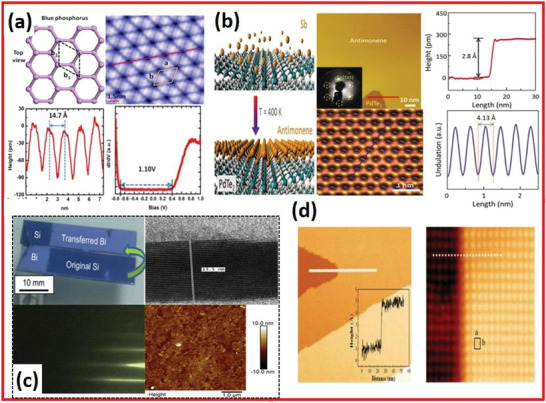
a) Blue phosphorene and its corresponding STM image. Reproduced with permission.^[^ ^87^ ^]^ Copyright 2016, American Chemical Society. b) TEM and AFM image of single‐layered antimonene. Reproduced with permission.^[^ ^89^ ^]^ Copyright 2017, Wiley‐VCH. c) Morphological presentations of bismuthine grown on silicon films and its corresponding images. Reproduced with permission.^[^ ^91^ ^]^ Copyright 2016, American Chemical Society. d) The left side image presents the topographic information at 1 V via epitaxial. The STM image at 0.6 V on the right side presents rectangular lattices. Reproduced with permission.^[^ ^92^ ^]^ Copyright 2017, Royal Society of Chemistry.

The yield of a high‐quality epitaxial layer utilizing traditional epitaxy growth methods requires the substrate and epitaxial layer to possess the same geometry and very nearer lattice constants. Koma and co‐workers presented van der Waals epitaxy to create 2D materials with vertical heterostructure.^[^ ^8^, ^86^ ^]^ In this method, van der Waals epitaxy employs substrates without introducing dangling bonds on their surfaces. The layers of epitaxial layers are directly connected to the substrates by weak forces, which support the epitaxial growth of 2D layered materials that develop crystalline symmetry to the substrate. This may also be possible when there is a difference of almost 50% in lattice constant without any strain in the epitaxial layer. Ji and co‐workers^[^ ^25^ ^]^ used van der Waals epitaxy to obtain layered antimonene polygons on KMg_3_(AlSi_3_O_10_)F_2_ substrates. A tube furnace with two‐zone is shown in **Figure** **24**a. The commercial powder was heated to 660 °C in the first T1 zone during the generation of antimony vapor. The schematic presentation of van der Waals epitaxy is shown in Figure 24b. The fluorophlogopite mica substrates were installed in the downstream area at *T*_2_ = 380 °C. The preparation method was kept running for 60 min, and the tube furnace stepped down to normal temperature. The thickness, crystal orientation and doping amount of the 2D group VA materials can be accurately controlled by MBE in Figure 24c,d. In particular, P4 atoms were precipitated in the form of vapors just on top of the Au (111) substrate by utilizing black phosphorus. Single‐layer phosphorous with the regular hexagonal morphology was generated during deposition and annealing.^[^ ^87^ ^]^ High‐resolution scanning tunneling microscopy (HRSTM) revealed that the single phosphorus layer was extremely organized for each black core encircled by six triangles, as illustrated in **Figure** **25**a. There was around 14.7 Å distance around dark centers on average. P4 was found to have a novel type of 2D semiconductor, as evidenced by the 1.10 eV bandgap observed for just a single layer of P4. The theoretical predictions provided by Zhu and Tománek were in agreement with the data.^[^ ^88^ ^]^ The new style for 2D phosphorus with such a thick “zigzag ridge” morphology was comparable to silene‐like a multilayer honeycomb pattern, as anticipated by DFT calculations.

2D antimony was also fabricated like 2D phosphorus by the implication of the MBE approach. On a stable chemical multilayer PdTe_2_ substrate, a monolayer of antimonene having uniform alignment and ordering grid can grow.^[^ ^89^ ^]^ The creation of the 2D antimony film is influenced by the modest surface lattice (4.01 Å) constant discrepancy, which is lesser than 2.3%. The emerging antimony film has a honeycomb lattice structure and is extremely organized and symmetrical. The height of antimony is measured to be 2.8 Å (Figure 25b). The 2D monolayer antimony's tunable bandgap suggested that it might find applications in electrical and photovoltaic devices. The MBE approach recognizes silicon as the most appropriate substitute substrate for the synthesis of monolayered 2D group VA semiconductors.^[^ ^3^, ^90^ ^]^ The silicon surface should be thoroughly cleaned before the deposition of monolayers of 2D materials. The Silicon substrate was first cleaned by soaking it in diluted hydrofluoric acid to eliminate the ordinary oxides. Then, the silicon substrate was put in a high vacuum at a high temperature for 20 minutes to stop the secondary oxidation process. For instance, by applying the MBE approach, researchers have generated full‐wafer monocrystalline bismuth films with a wide range of thicknesses (4 to 50 nm) on silicon substrates (Figure 25c).^[^ ^91^ ^]^ Tellurene was also newly grown‐up on the graphene‐based 6H‐SiC substrate using MBE technology.^[^ ^92^ ^]^ Its lattice is rectangular and is close to the theoretical lattice constant (Figure 25d). The bandgap of tellurium decreases as the layer thickness increases.

It is suggested that at the atomic scale level, the growth of 2D group VA materials can be controlled using slow deposition rates, but the complexity of MBE may limit its wider application.

#### 4.2.3 Solvothermal Method

**Figure 26:**
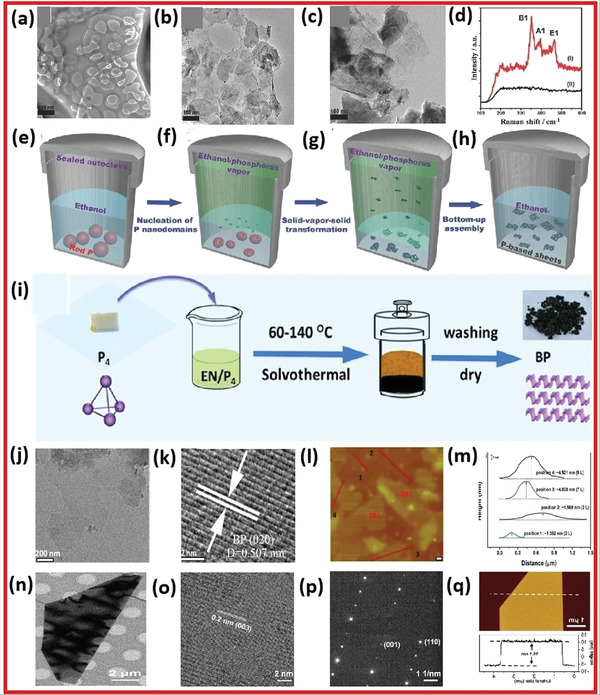
The morphological patterns of (bulk) phosphorus under various reaction times a) 2 h, b) 12 h, and c) 24 h. d) Raman spectrum. Schematics diagram of the transformation from phosphorus to phosphorene: e) sublimation, f) nanomaterial formation, g) nanosheet formation, and h) final product (in ethanol). Reproduced with permission.^[^ ^93^ ^]^ Copyright 2015, Wiley‐VCH. i) Synthetic solvothermal method of BP. j) TEM image of the BP nanosheets. k) HRTEM image and l) AFM image of BP flakes. Reproduced with permission.^[^ ^94^ ^]^ Copyright 2018, National Academy of Sciences. n) TEM image of tellurium. o) HRTEM image. p) SAED pattern. q) AFM image and corresponding height profile. Reproduced with permission.^[^ ^95^ ^]^ Copyright 2018, American Chemical Society.

The solvothermal method is a popular technique for creating nanomaterials. According to the literature, the solvent‐thermal strategy is a consistent method to develop BP nanosheets. In particular, a large yield of 30% nanosheets of BP was synthesized at about 400 °C (**Figure** **26**a).^[^ ^93^ ^]^ The homogeneous 2D nanocrystals were produced after vacuum drying, which was identified as having about 8 layers of phosphorus atoms as presented in Figure 26b–d. The 040 and 002 planes of perpendicular BP had correspondingly diffraction patterns of 0.27 nm and 0.23 nm. The developed BP nanosheets are ideal for use as the anode material in lithium‐ion batteries because of their good electrochemical characteristics.

Tian and co‐workers^[^ ^94^ ^]^ used bulk white phosphorus to prepare BP with a few atomic layers using a solvothermal method. Additionally, the harvest of BP is amplified by increasing the solvothermal reaction temperature (between 60 and 140 °C) as presented in Figure 26i. The synthesized BP comprises 2 to 28 atomic layers and ranges in thickness from 1 to 15 nm (Figure 26j–l).

For the synthesis of 2D tellurium by the solvothermal method, sodium tellurite (Na_2_TeO_3_) is used as the tellurium source, with hydrazine hydrate (N_2_H_4_·H_2_O) as the reducing agent.^[^ ^95^ ^]^ As shown in Figure 26n–q, the TEM, HRTEM, and AFM investigations demonstrated that this technique could achieve tellurene nanosheets with a consistent size of approximately 15 µm. The results revealed tellurene had uninterrupted lattices with a lattice parameter of 2, which correlates to the Te (003) plane. Furthermore, by manipulating reaction conditions, the thickness can be varied from single layer to 10 nm. The resulting 2D nanosheets are extremely stable. In summary, the reaction temperature, raw materials, solvents, and surfactants impact the yield and shape of 2D materials in the solvothermal process.

## 5 Functional Nanostructures

2D group VA materials can be further modified to create a wide range of functional nanostructures for various applications. Hybrid heterostructures, atom molecule doping, and surface functionalization of 2D group VA materials are all possible approaches. Particularly, synergistic effects would be the outcome in hybrid materials with original and enhanced physicochemical characteristics.

Surface modification is a proper method for enhancing the effectiveness of 2D group VA materials. Despite significant progress in studying BP‐modified nanostructures, the main limitation of BP implications is its degradation behavior when exposed to ambient environments for long periods. Perpendicular to the BP surface, the interaction involving oxygenation and lone‐pair electrons produces PO*_x_* species, which cause BP degradation. Beyond BP, 2D group VA materials like single‐ to few‐layer arsenene, antimonene, and bismuthene are promising candidates with superior physical and chemical properties. They have special applications in various fields due to their unique in‐plane anisotropic structure, high charge‐carrier mobility, and tunable direct bandgap. Various functionalization methods, e.g., electrostatic functionalization, van der Waals functionalization, and covalent functionalization are used to modify 2D group VA semiconductors before practical applications. Lewis acids, for example, alkali ions, transition metal ions, and organic molecules can interact with 2D material surfaces. Positively charged adsorbates spontaneously interact with negatively charged nanomaterials in electrostatic functionalization. Associations of 2D materials with capping layers, such as hexagonal boron nitride, graphene, etc., belong to van der Waals functionalization. Surface modification can improve the stability of 2D materials while preserving and boosting their long‐term property.

Surface modification of 2D group VA materials primarily entails physical and chemical steps, for instance, ball milling, thermal evaporation, assembling, vacuum deposition, solvent evaporation, impregnation, spin coating, self‐assembly, photoreduction, chemical reaction, focused laser‐induced oxidation, phase conversion, etc. These methods are commonly used in developing a wide range of optical, optoelectronic, and electronic devices, including Li‐ion batteries, Na‐ion batteries, junction FETs, memory devices, photodetectors, and so on.

## 6 Applications

### 6.1 FET

FET, an essential component of up‐to‐date integrated circuits, is among the most prominent semiconductor applications. There are no dangling bonds, unwanted links with phonons, or the formation of interface states in FETs fabricated via 2D materials. Leakage current can be reduced in FETs with 2D semiconductor channels because all electrons are constrained in nanoscale channels and thus homogeneously impacted by the gate voltage.^[^ ^94^ ^]^ Semiconducting group VA crystals have demonstrated their utility as semiconductor layers. As FET channel materials, BP has two key benefits over graphene and MoS_2_: elevated on/off current ratio for efficient switching and charge carrier concentration for fast operation.

**Figure 27:**
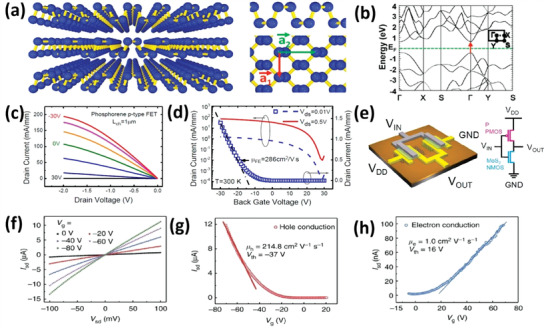
a) Overview of phosphorene with various viewpoint. b) Band structure of phosphorene monolayer (HSE06 band structure). c) Output curves of phosphorene‐based (p‐type) transistors. d) Transfer curves. Reproduced with permission.^[^ ^4a^ ^]^Copyright 2014, American Chemical Society. e) Phosphorene‐based inverter configuration. Reproduced with permission.^[^ ^95^ ^]^ Copyright 2018, American Chemical Society. f) Phosphorene‐based FET device: *I*_sd_–*V*_sd_ evaluations at various gate voltages (0–80 V). Isolated transfer curves for g) hole and h) electron conduction. Reproduced with permission.^[^ ^96^ ^]^ Copyright 2015, Springer Nature.

Liu and co‐workers^[^ ^4a^ ^]^ examined the photoelectrochemical characteristics of exfoliated nanoflakes of phosphorene, as shown in **Figure** **27**a. In Figure 27b, they performed ab initio DFT simulations to regulate the structural symmetry and interfacial adhesion interrelations of high‐volume bulk phosphorus and 2D phosphorene. The bandgap is influenced by layer number and strain inside layers, according to their theoretical results. The visible‐wavelength photoluminescence peak observed in monolayered phosphorene indirectly favors the tunable bandgap predicted by theory. Phosphorene's transport behavior shows significant anisotropy, which can be attributed to the layers' unique ridge structure. The Schottky barrier height (0.21 eV) for hole tunneling at the junctions between Ti metal contacts and phosphorene can explain the overall device behavior. In Figure 27c,d, few‐layered phosphorene‐based p‐type transistors show high field‐effect mobility of 286 cm^2^ V^−1^ s^−1^ and a current on/off ratio of over 10^4^ at room temperature. An inverter was also built based on a phosphorene transistor and a MoS_2_ transistor, allowing for heterogeneous integration of phosphorene for future electrical applications. Xiang and co‐workers^[^ ^96^ ^]^ designed phosphorene‐based FET via surface doping by Cs_2_CO_3_ and MoO_3_. The ambipolar phosphorene FETs presented charge carrier mobility up to ≈27 cm^2^ V^−1^ s^−1^ using 10 nm Cs_2_CO_3_ surface modification. Figure 27e illustrates the configuration of the phosphorene‐based inverter.^[^ ^95^ ^]^ On the other hand, MoO_3_ doping presented massive doping properties for FET devices and the mobility was about ≈200 cm^2^ V^−1^ s^−1^, as shown in Figure 27f–h. Their findings indicate a straightforward technique for doping phosphorene, which can successfully modify the ambipolar properties to create high‐performance electronic devices.^[^ ^96^ ^]^

**Figure 28:**
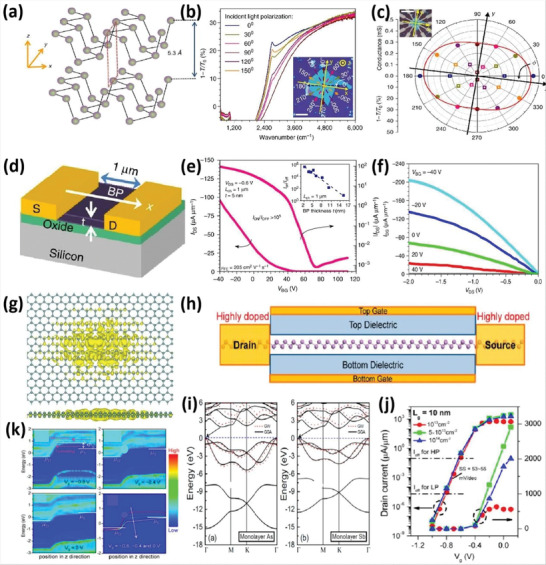
a) BP crystalline structure. b) Polarization‐resolved infrared relative extinction spectra. c) The same six directions were measured. d) Phosphorene‐based FET. e) Transfer characteristics of FET. f) Output characteristics. Reproduced with permission.^[^ ^3b^ ^]^ Copyright 2014, Springer Nature. g) Multilayered arsenene (top and side viewpoints). h) Schematic presentations of arsenene‐based device. i) The band structures of multi‐layered 2D group VA members (arsenene and antimonene). j) Transport characteristics. k) The conduction bands are outlined. Reproduced with permission.^[^ ^27^ ^]^ Copyright 2017, American Chemical Society.

The photodetector's behaviors of phosphorene devices were also investigated. The crystalline structure of BP is shown in **Figure** **28**a. The six directions were measured along with current conductivity as shown in Figure 28b–d. The transfer characteristics and output characteristics of BP‐based FET are presented in Figure 28e,f.^[^ ^16^ ^]^

Wang and co‐workers^[^ ^27^ ^]^ investigated the monolayer antimonene and arsenene‐based FET devices with high charge carrier mobility. In 10 nm arsenene FETs, the carrier mobilities are 21 cm^2^ V^−1^ s^−1^ and 66 cm^2^ V^−1^ s^−1^ for electrons and holes, correspondingly. They have claimed that the improved gate electrostatics and flat surface of 2D group VA semiconductors make the balanced and stable channel materials for next‐generation electronics. Arsenene has been fabricated very recently, and the schematic presentations of arsenene‐based devices are shown in Figure 28g,h. The bandgaps of multi‐layered antimonene and arsenene are in the range of 2.38 and 2.47 eV, as illustrated in Figure 28i. The electrical performances were changed when the doping concentrations were different, as shown in Figure 28j.^[^ ^46^ ^]^ According to the GW‐Bethe‐Salpeter equation, the optical energy bandgaps for multi‐layered arsenene and antimonene are 1.6 and 1.5 eV, and charge carrier binding affinities are 0.9 and 0.8 eV, correspondingly. The charge carrier mobility in multi‐layered antimonene is higher as compared to multi‐layered arsenene (150/510 cm^2^ V^−1^ s^−1^ and 21/66 cm^2^ V^−1^ s^−1^ correspondingly). Multi‐layered antimonene and arsenene are desirable for electronic and optoelectronic device applications due to their documented great stability under ambient environments.

**Figure 29:**
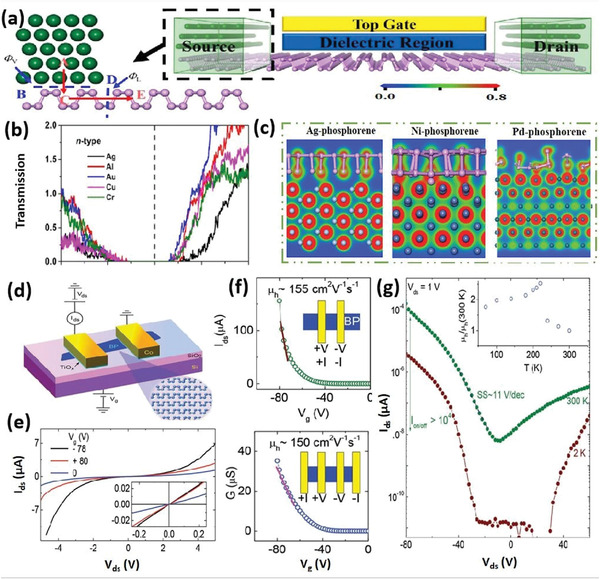
a) Multilayered phosphorene‐based FET. b) Transmission properties. c) Contour plots of total electron distribution of multi‐layered phosphorene with Ag electrode, multilayered phosphorene with Ni electrode, and multi‐layered phosphorene with palladium electrode. The various lights (purple, light blue, dark blue, yellow, and green balls) showed specific atoms (P, Ag, Ni, and Pd atoms, correspondingly). Reproduced with permission.^[^ ^97^ ^]^ Copyright 2015, American Chemical Society. d) The phosphorene‐based FET device (ferromagnetic contacts). e) Output curves of the device. Inset: amplified low distortion linear area. f) *V*_ds_ evaluations at 1 V at mobility vale of *µ*_h_ ≈ 155 cm^2^ V^−1^ s^−1^ and yielding *µ*_h_ ≈ 150 cm^2^ V^−1^ s^−1^ at the four‐probe system. g) Transfer curves (300 K and 2 K). Reproduced with permission.^[^ ^98^ ^]^ Copyright 2015, Wiley‐VCH.

Pan and co‐workers^[^ ^97^ ^]^ examined a multi‐layered phosphorene‐based FET device with various metal‐based electrodes, as presented in **Figure** **29**a. They investigated the interfacial characteristics between the metal electrode and phosphorene surface via combined ab initio electronic structure calculations with quantum transport simulations. Strong interactions could be formed between all the metals and multi‐layered phosphorene. Unlike the energy structure calculations, the quantum transport simulations are more reliable in describing Schottky barrier height (SBH) due to the interactions between phosphorene and metal electrodes. All of the examined metal surfaces and multi‐layered phosphorene develop robust contacts. In modeling SBH, the quantum transport models are more precise when significantly compared to predictions of the energy structure. The simulations match well with the results of the experiments.

According to quantum transport simulations, multilayered phosphorene and various electrodes (Au, Cu, Cr, Al, and Ag) create n‐type Schottky contacts with electron SBHs of 0.30, 0.34, 0.37, 0.51, and 0.52 eV, correspondingly. Moreover, Figure 29b,c show multi‐layered phosphorene with Ti, Ni, and Pd electrodes which produce P‐type Schottky contacts with hole SBHs of 0.30, 0.26, and 0.16 eV, correspondingly. Surprisingly, Au produces the smallest electron SBH‐mediated n‐type Schottky contact with multi‐layered phosphorene. The practical SBH data strongly indicates that the findings can be used as a guide for future multi‐layered phosphorene‐based electrical device design when deciding which metal electrode to use.

Kamalakar and co‐workers^[^ ^98^ ^]^ also explored the 2D BP‐based FET, which has ferromagnetic channel contacts as shown in Figure 29d. Utilizing TiO_2_/Co contacts, low surface resistances with ambipolar behavior could be produced with low SBH. The hole conduction regime demonstrated an ambipolar transistor with an on/off current ratio of > 10^4^ and an on‐state current of 150 µA µm^−1^ (Figure 28e). Because of the reduced bandgap of multilayer BP, the ambipolar property is prominent where hole mobility is about 155 cm^−2^ V^−1^ s^−1^ (Figure 29f). The models anticipate the detection of magnetoresistance (MR) utilizing TiO_2_/Co tunnel contacts over a broad range of gate lengths and durations. Furthermore, the gate voltage can tune the MR, which changes the contact resistance and carrier density in BP. Incorporating these ferromagnetic materials can eliminate the conductance incompatibility issue that prevents effective spinning insertion and offers the opportunity for spintronic devices made of 2D group VA semiconductors.

### 6.2 Photodetectors

**Figure 30:**
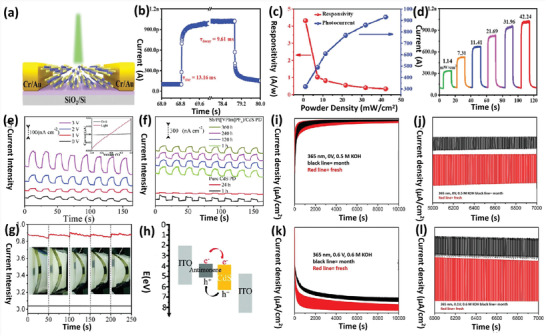
a) The photodetector based on Sb_2_Se_3_ flake. b) The photo response at 42.24 mW cm^−2^ laser intensity. c) The photocurrent and responsivity presentations. d) Current versus time.Reproduced with permission.^[^ ^101^ ^]^  Copyright 2017, John Wiley and Sons. e,f) The photo response under 532 nm laser intensity at 2 V. g) The stability test of the antimonene‐based photodetector. h) The schematic mechanism for the flexible photodetector. Reproduced with permission.^[^ ^80^ ^]^ Copyright 2020, Wiley‐VCH. i–l) The bismuthine‐based photodetector under 365 nm light (earlier and after 30 d) and photo response characteristics. Reproduced with permission.^[^ ^64^ ^]^ Copyright 2017, American Chemical Society.

The rise in global power consumption and population over the last decade has stimulated the improvement of energy‐efficient and long‐term optical energy detection methods.^[^ ^99^ ^]^ Photodetectors convert photons into electrical signals and are critical in various fields, including security, environmental and industrial process control.^[^ ^100^ ^]^ UV photodetectors are one of the most promising energy detection systems, with potential applications in human daily life and military defense. Furthermore, UV radiation is sensitive to the human body. It can cause a range of illnesses, including cataracts and skin cancer. 2D antimonene has high charge carrier mobility, excellent stability, and tunable direct bandgap, which have fascinated researchers to fabricate unique/novel photodetectors. BP can be used to detect infrared light. However, fabricating highly efficient IR photodetectors with long lifetimes is difficult due to BP's poor air stability and high contact resistance with metals. Zhao and co‐workers^[^ ^80^ ^]^ used a CVD method to create 2D ultrathin Sb_2_Se flakes (**Figure** **30**a). The thickness‐dependent 2D Sb_2_Se_3_ flake's band structure exhibits a broad absorption band of 300 to 1000 nm (UV to NIR), implying that it could be used in wideband photodetection. The as‐fabricated Sb_2_Se_3_ photodetector exhibited broad photoresponse ranging from ultraviolet to NIR, with a quick response time (*τ*_rise_: 13.16 ms and *τ*_decay_: 9.61 ms) with an anisotropic ratio of 2.5 @ 532 nm (Figure 30b–d).

Xiao and co‐workers^[^ ^101^ ^]^ adopted a polymer ionic liquid‐assisted LPE technique to fabricate few‐layered antimonene, and the photocurrents of antimonene‐based devices under various bias voltages are depicted in Figure 30e.^[^ ^101^ ^]^ The photocurrent density enlarged directly in the range of 20 to 160 nA cm^−2^ at 0 to 3 V. The flexible photodetector device had an on/off current ratio of 26.8 under a bias of 1 V. The stability test of the antimonene‐based photodetector is shown in Figure 30f. The reference photodetector was made of CdS QD. The photocurrent density of the reference photodetector was reduced by more than 80% within 24 h. They also evaluated the mechanical stability of the flexible photodetector based on monolayered antimonene at various bending states, as shown in Figure 30g. Figure 30h shows a schematic mechanism for the flexible photodetector. In a vacuum, the antimonene's valence and conduction bands are 5.3 and 3.7 eV, respectively.

Uniform‐sized bismuthene QDs via the LPE method were prepared.^[^ ^64^ ^]^ The optical absorption spectra of bismuthene QDs revealed a wide range of absorption from 200 to 600 nm. Photoresponse stability of the photodetector under 365 nm illumination before and after one month is presented in Figure 30i,j. The photo response behavior in 0.1 m KOH with 0.6 V bias potential and self‐driven behavior in 0.5 m KOH without bias potential is illustrated in Figure 30k,l. The light at 475, 520, and 550 nm can activate the photo response characteristic of the bismuthene QD‐based photodetector. The resulting bismuthene QDs show promise for potential applications in UV–visible photodetectors.

**Figure 31:**
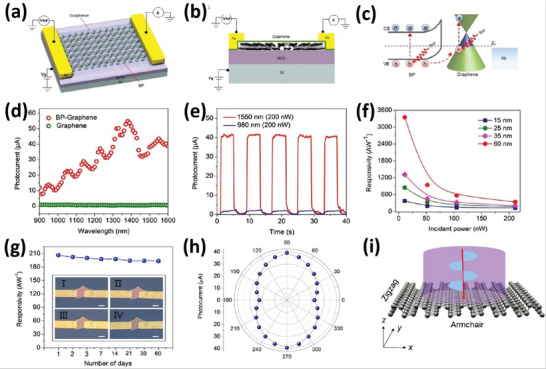
a,b) Schematic illustration of the phosphorene‐based heterostructured photodetector. c) The band diagram of the device. d) The photocurrent is dependent on the excitation wavelength, which is in the range of 900 to 1600 nm. e) Photoresponse of the heterostructure of graphene–phosphorene. f) The responsivity of a heterostructure of graphene‐phosphorene. g) The responsivity was evaluated in the air condition. h) Photocurrent as a function of polarization angle. (i) BP crystal lattice all along the x, y, and z‐axis. Reproduced with permission.^[^ ^102^ ^]^ Copyright 2017, American Chemical Society.

A phosphorene heterojunction‐based detector featuring extremely high responsiveness and long‐term durability at IR wavelengths was fabricated by Liu and co‐workers (**Figure** **31**a–c).^[^ ^102^ ^]^ The upper surface of graphene in their device serves as a transportation layer and an encapsulating layer. Photoexcited electron–hole pairs produced in phosphorene are separated and infused into graphene under illumination. Figure 31d,e shows the photo responsiveness of the photodetector (3.3 × 10^3^ A W^−1^) and a rise time of roughly 4 ms at 1550 nm. However, thicker phosphorene flakes exhibited stronger photo responses (Figure 31f). The heterostructure photodetector (graphene–phosphorene) also shows increased photosensitive efficiency, ultrafast charge transfer, polarization response, as well as durability. The 2D group VA heterostructure is a powerful photodetection platform that can maintain high performance in the IR wavelength regime, making it useful for remote sensing, biomedical imaging, and environmental control.

**Figure 32:**
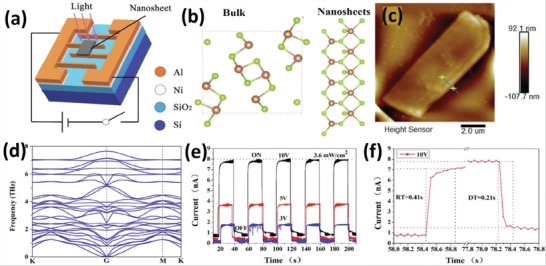
a) The Sb_2_Se_3_ single nanosheet detector structure. b) Bulk and monolayered Sb_2_Se_3_. c) AFM image of Sb_2_Se_3_. d) The phonon dispersion of monolayered Sb_2_Se_3_. e) I–T curve under the influence of a 405 nm laser. The light intensity value was 3.6 mW cm^−2^, and at various voltages (3, 5, and 10 V). f) The rising and decaying characteristics for a bias voltage of 10 V. Reproduced with permission.^[^ ^103^ ^]^ Copyright 2017, Wiley‐VCH.

Wang and co‐workers^[^ ^103^ ^]^ fabricated Sb_2_Se_3_ nanosheet‐based photodetector (**Figure** **32**a–c). When evaluating absorption spectrum spectra, the bandgap of the nanosheets is estimated to be 1.55 eV. According to an ab‐initio study of the electronic band structure. the bandgap is estimated to be 1.58 eV, which is in good accordance with experiment data. The device demonstrated a remarkable response to 405 nm light at 3.6 mW cm^−2^ (Figure 32e,f). Sb_2_Se_3_ nanosheets have potential applications in flexible optoelectronic devices with low cost and good stability. Exhaustive efforts have been made and researchers should continue to develop photodetectors with distinct advantages over traditional technologies.

### 6.3 Memristors

Researchers have been fascinated by the emerging neuromorphic computing system over the years because of its exclusive aspect of integrated processing and memory units, which can exceed the conventional von Neumann computing.^[^ ^104^ ^]^ Brain‐inspired electronics provide a potential framework to address traditional digital machines' performance and energy constraints. Current advancements have been made by mimicking highly adaptive biological strategies. Memristors, which can mimic biological neurons and synapses, have been thought to be building blocks for an artificial neural computing system.^[^ ^105^ ^]^ Synaptic functions including short‐term plasticity (STP), inhibitory postsynaptic current (IPSC), excitatory postsynaptic current (EPSC), long‐term depression (LTD), and long‐term potentiation (LTP), etc. have been successfully simulated in various 2D material based devices.^[^ ^106^ ^]^

**Figure 33:**
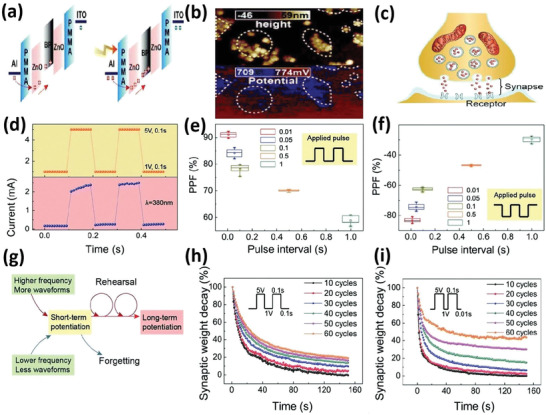
a) Energy band diagram of the device with application of external bias in the dark and energy band diagram under light illumination. b) KPFM image. c) The schematic illustration of the synapse. d) Application of 380 nm pulses. e) PPF. f) PPD. g) STP to LTP and the learning diagram. h) The synaptic weight decays over time. (0.1 s interval). i) The synaptic weight decays over time (0.01s interval). Reproduced with permission.^[^ ^104b^ ^]^ Copyright 2018, Royal Society of Chemistry.

Ren and co‐workers^[^ ^104b^ ^]^ fabricated heterostructured ZnO–phosphorene nanoparticles‐based memristor. The production of oxygen vacancies conducting filament is supposed to be the key to the device's light‐modulated characteristic, which would be believed to arise from the photoelectric and photogate effects. The energy band diagram of the device with the application of external bias in the dark is shown in **Figure** **33**a. The Kelvin probe force microscopy (KPFM) image and energy band diagram under light illumination is presented in Figure 33b,c. Additionally, the memristor can simulate crucial synaptic functions like PPF, SRDP, STDP, and the transition from STP to LTP, and optically controlled plasticity has been achieved (Figure 33d–f). They also examined how variable factors, such as pulse width, time interval, and potentiating voltage, affect the device conductance modulation. By using pulse trains, the synaptic weight decays over time (Figure 33g–i). Furthermore, it is demonstrated that the heterostructured ZnO–phosphorene nanoparticles‐based memristor has excellent thermal stability.

**Figure 34:**
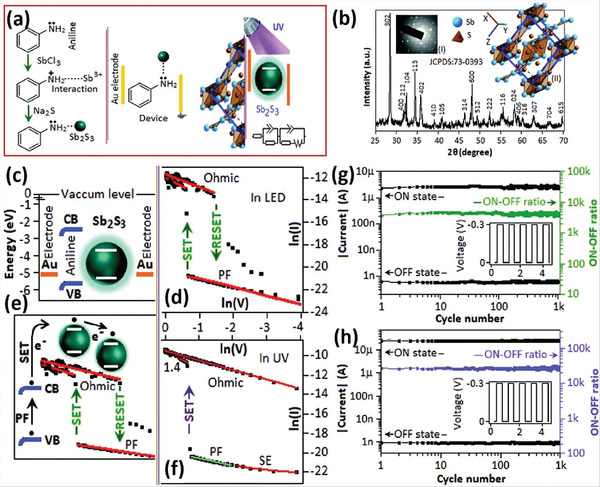
a) The synthesis of aniline‐controlled antimony sulfide nanoparticle. b) XRD spectrum of antimony sulfide and SAED pattern. c) The device's schematic energy diagram. d) The transport mechanism displays the Poole‐Frankel (PF) and Ohmic conduction for the OFF‐state and ON‐state, respectively. e) A charge transfer process. f) The device's transport mechanism under UV irradiation, with the Schottky and P–F emission models for the OFF‐state and the Ohmic conduction mechanism for the ON‐state. g) The device's endurance under research lab luminance and h) UV exposure. Reproduced with permission.^[^ ^107^ ^]^ Copyright 2021, Royal Society of Chemistry.

Perla and co‐workers^[^ ^107^ ^]^ reported the complexation‐mediated synthesis of aniline stabilized antimony sulfide as shown in **Figure** **34**a. The XRD pattern confirmed the successful formation of antimony sulfide (Figure 34b). The energy diagram and the IV curve of the device exhibited a unipolar characteristic with an on/off current ratio in the order of 10^3^. The on‐state followed the Ohmic conduction mechanism. In contrast, the off‐state followed the Poole‐Frenkel (P‐F) emission model (Figure 34c,d). Figure 34e shows the schematic illustration of the charge transfer process. Figure 34f illustrates the transport mechanism of the device under the UV irradiation condition, where the Ohmic conduction mechanism was followed for the on‐state, and the Schottky and P–F emission models were fitted for the off‐state. Figure 34g,h shows the endurance study of the memristor under laboratory illumination and UV irradiation conditions. The IV characteristics exhibited a bipolar‐type switching behavior with the on/off current ratio in the order of 10^4^ under the UV irradiation condition. The cumulative distribution probability study of the device suggested a moderately stable performance under LED and UV irradiation conditions. The frequency study demonstrated that the aniline stabilized antimony sulfide‐based device was more conductive under the UV irradiation condition in the low‐frequency region.

### 6.4 Sensors

The practical fabrication of 2D group VA semiconductors employing top‐down techniques like LPE or bottom‐up techniques like epitaxial growth has made these materials increasingly attractive for sensor design.^[^ ^54^, ^108^ ^]^ The 2D phosphorene, antimonene, bismuthene, and arsenene can be used as important structural components to design high‐quality electrochemical sensors, which are used for the detection of widespread chemicals molecules. Researchers can easily analyze gaseous and solid‐state specimens because of their good characteristics.

Although the biochemical influence of a 2D group VA semiconductor is still largely unknown, this should not be disregarded. It was discovered that the single‐layered arsenene was quite poisonous. Investigation of these materials is difficult because layered 2D monoelemental and nanocomposite materials could deteriorate in environmental settings. Both covalent and non‐covalent organic functionalization enabled corrosion protection is very appealing for innovative sensing device design.

**Figure 35:**
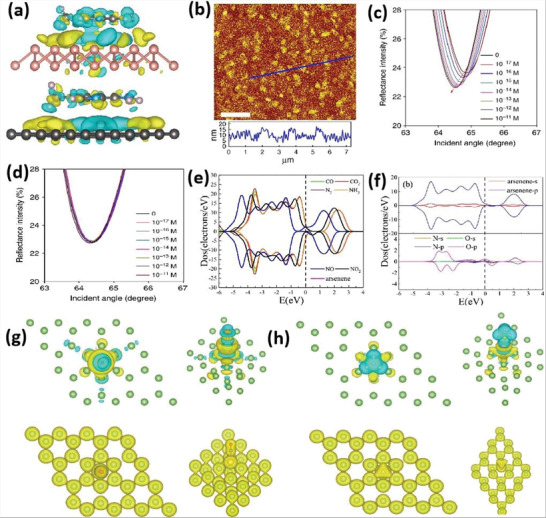
a) Corner viewpoints of charge density difference between antimonene and graphene nucleobases. b) AFM image of antimonene on the gold substrate with a 2 µm scale. c,d) The SPR spectrum presents 10^−17^ to 10^−11^ m miRNA‐21 concentrations. Reproduced with permission.^[^ ^109^ ^]^ Copyright 2019, Springer Nature. e,f) NH_3_ adsorption on Pt‐decorated monolayered arsenene to check the change in electronic densities and total electron density schemes. g,h) The simulation data of Pt‐decorated monolayered arsenene. Reproduced with permission.^[^ ^110^ ^]^ Copyright 2020, Elsevier.

Xue and co‐workers^[^ ^109^ ^]^ fabricated antimonene assisted surface plasmon resonance (SPR) sensor (**Figure** **35**a,b), which was used for the quantitative cancer diagnosis. The antimonene‐assisted SPR sensor was used to observe the miRNA‐21 and miRNA‐155. The maximum specificity of miRNA identification related to direct detection and monitoring of miRNA concentrations takes a LOD of 10 Am. Utilizing Au nanorods intensification, Figure 35c displays SPR bands for miRNA‐21 varying concentrations (ranging from 10^−17^ to 10^−11^ m). The arrow represents the SPR angle displacement. Figure 35d displays SPR bands with no Au nanorods (at miRNA‐21) with concentrations between 10^−17^ to 10^−11^ m. Significantly, computational and practical examinations were done on the signal magnification property of Au nanorods and the interface between single‐layered antimonene and DNA (ssDNA/dsDNA).

Thus, the suggested biosensor marks the very first technique for therapeutically relevant nucleic acid detection employing antimonene materials. It offers a tremendous possibility for the creation of lab‐on‐chip systems. Future studies of the high throughput and specificity are crucial for the therapeutic and practical applications of the antimonene‐based SPR sensor to be realized, including the early diagnosis of cancer and point‐of‐care systems.

Co, Cu and Pt functionalized arsenene^[^ ^110^ ^]^ has been fabricated as sensors for detecting various gaseous molecules. The adsorption mechanism of a Pt‐modified mono‐layered arsenene towards gas molecules was further investigated using band structures, charge transport, and the difference between electron density. The findings indicate that the adsorption of gas molecules on the Pt‐modified monolayered arsenene is more promising than pristine arsenene. The electron density variation strategies for platinum‐modified arsenene systems with adsorption of CO and NH_3_ molecules are shown in Figure 35g,h. According to the computed adsorption energies, the sulfur dioxide (SO_2_) molecule has the strongest adsorption intensity on the surface of Pt‐modified arsenene. As a result, compared to other molecules, Pt‐modified arsenene was the most sensitive for detecting SO_2_. According to the proposed hypothesis, the Pt‐modified arsenene as shown in Figure 35e,f are potential materials for chemical sensors to identify dangerous gas molecules in the environment.

### 6.5 Batteries

2D group VA materials have recently fascinated the attention of many researchers owing to their interesting electronic properties.^[^ ^2^, ^53^, ^59^ ^]^ 2D materials with different chemical and physical properties can exceed their bulk counterparts as electrode materials for rechargeable batteries. According to simulation and experiment studies, BP has a powerful in‐plane covalent bonding infrastructure, but weak van der Waals interfacial exchanges and is illustrated as an encouraging bonding material for batteries with good binding strength.^[^ ^111^ ^]^

**Figure 36:**
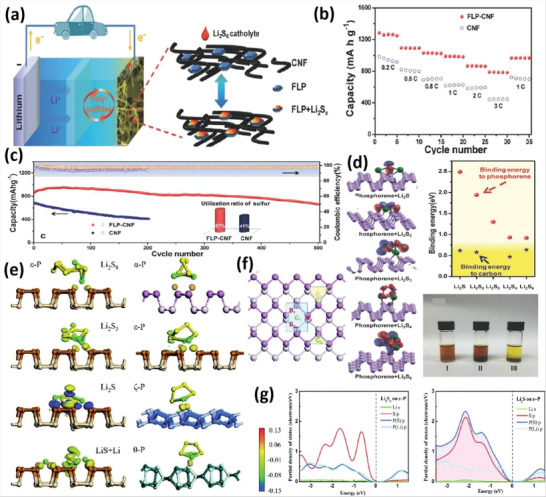
a) Li–S battery. b) Capacity concerning cycle number. c) Electrochemical long cycling. d) Lithium polysulfide adsorption's hypothetical calculations and experimental data. Reproduced with permission.^[^ ^111a^ ^]^ Copyright 2016, Wiley‐VCH. e) Differences among various electron densities. Reproduced with permission.^[^ ^111c^ ^]^ Copyright 2020, American Chemical Society. f) Initial adsorption sites on BP. Reproduced with permission.^[^ ^111a^ ^]^ Copyright 2016, Wiley. g) The partial density states near the Fermi level. Reproduced with permission.^[^ ^111c^ ^]^ Copyright 2020, American Chemical Society.

Li and co‐workers^[^ ^111a^ ^]^ demonstrated that phosphorene is a potential candidate for the lithium polysulfide catholyte (**Figure** **36**a). It works as an electrocatalyst and also has the characteristic of a polysulfide immobilizer for lithium‐sulfur batteries. Phosphorene's capability to control polysulfides makes it a very useful and inspiring skill to test at various current densities and extend the cycle life of lithium–sulfur cells, as presented in Figure 36b,c. The numerical simulations and empirical studies of the lithium polysulfide deposition are shown in Figure 36d. Li and co‐workers^[130]^ investigated eight allotropes of phosphorene *α*‐P, *β*‐P, *γ*‐P, *δ*‐P, *ζ*‐P, *η*‐P, and *θ*‐P for lithium–sulfur batteries as fixing materials. Results proved that all allotropic forms of phosphorene have an excellent ability to bind with lithium–sulfur. Their findings also revealed that the concentration of phosphorene reduces polarization, speeds up the redox reaction, and enhances sulfur utilization in rechargeable batteries. Figure 36e presents differences among electron densities^[^ ^111c^ ^]^ and Figure 36f shows the initial adsorption sites on phosphorene.^[^ ^111a^ ^]^ Moreover, the partial density states near the Fermi level can be seen in Figure 36g.^[^ ^111c^ ^]^ This study will energize the interest of researchers to start investigating the basic science and implementations of 2D group VA materials in energy storage devices and catalysis.

## 7 Future Prospect

In 2D group VA semiconductors, phosphorene has been mostly investigated. It has intriguing features, for example, tunable bandgap, high carrier mobility, negative Poisson's ratio, and anisotropic properties. External influences like defect introduction, chemical functionalization, heteroatom doping, electric field, heterostructure synthesis, and strain can significantly alter these properties. However, phosphorene has an Achilles’ heel because of its quick oxidation tendency and light‐induced breakdown of exfoliated nanoflakes in the presence of damp circumstances. More than a few solutions for resolving this problem have been proposed, and the steadiness mechanisms are investigated. Still, phosphorene's real‐world pertinence is in its early stages. To speed the realization of phosphorene‐based devices, investigators must focus on reducing oxidation and improving long‐term resilience under atmospheric conditions.

Recent studies on arsenene are anchored on DFT calculations. In experiments, extremely poisonous arsenic trioxide can be created during the preparation of 2D arsenene, limiting the experimental study of arsenene. Few‐layered arsenene on InAs can be manufactured via plasma‐assisted technique. Moreover, the fabrication of few‐layered arsenene for electrochemical applications has been achieved via an aqueous shear exfoliation approach. These findings give experimental proof aimed at effectively fabricating monoelemental arsenene. Moreover, it could open the door for more experimental studies of arsenene's exclusive characteristics. Quite a few theoretical investigations on antimonene have estimated various exotic characteristics and applications, from optoelectronic applications to energy storage and topologic aspects arising from the band structure. Since 2016, various experimental outcomes have brought all those predictions nearer to certainty. Antimonene initially demonstrated its environmental stability, and the heterostructure‐related potential uses will soon be expanded to encompass catalysis, biomedical engineering, and other sectors.

Bismuthene has gathered a lot of consideration because of strong spin–orbit coupling, quantum size effects, low carrier density, long mean free path, etc. Creating bismuthine‐engineered insulators will be predominantly significant. Bismuthene's electrical state differs greatly from that of its bulk 3D crystal. Bismuthene is a narrow‐band semiconductor due to the compression of the 2D lattice, which confines the electrons in the sheet. *α*‐bismuthene, the known 2D layered form of bismuth, is composed of a wavy structure in 2D hexagonal lattices like blue phosphorene, gray arsenene, and antimonene, except layered *β*‐bismuthene, which has been predicted but not yet empirically demonstrated. Bismuthine, on the other hand, has only an indirect and small bandgap up to 0.3 eV, which may limit its applications in electronic applications. Bismuthene's characteristics can be altered in a variety of ways, making it a versatile material. The bandgap of bismuthene is reduced when bismuthene layers are stacked on top of each other to form bilayers. More than two layers stacked on top of each other are thought to entirely close the bandgap, affecting the topological features of the 2D material. Strain and isoelectronic substitutional alloying can also be used to tune these topological and electronic features.

Remarkably, bismuthene is relatively stable in most conditions and is not difficult to grow into a large‐scale sample in a controlled manner. As a result, bismuthene can be used in more sophisticated devices with numerous production processes. There is a great deal of interest in studying the electrical transport properties of bismuthene. Certain research groups used PLD to create multilayer bismuthene on a centimeter scale in two different orientations (110) and (111). The thickness‐dependent bandgap transformation was achieved by modulating the processing temperature during the PLD process, resulting in a tiny bandgap of ≈0.2 eV in the 1 nm ultrathin bismuth film. It is worth noting that ultrathin (110) oriented bismuthene exhibits electric insulating properties, but few‐layer (111) oriented bismuthene exhibits layer‐dependent bandgap transition and a p‐type semiconductor characteristic with relative high carrier mobility of ≈220 cm^2^ V^−1^ s^−1^. Higher carrier mobility of 10^4^ cm^2^ V^−1^ s^−1^ is observed in a thicker bismuth film, indicating its potential for future development of electronic devices such as logic transistors. Point defects (i.e., atomic vacancies, atomic substitution, or interstitial atoms) can also be used to alter the electrical, magnetic and optical characteristics, as well as topological phase. Bismuthene sheets are stable at high temperatures even after the atomic alteration. The amount by which the characteristics vary in this approach is determined by both the energy of localized states and the strength of the coupling between point defects. Bismuthene can be used in a wide range of electronic applications, including spintronic devices, computation and data communications, quantum spin Hall insulating materials, and saturable absorbers. Including extra group VA monolayer arsenene and antimonene in a composite strategy to produce large‐gap quantum spin Hall systems could pave the way for a more systematic approach. One of the special and appealing aspects of phosphorene, arsenene, antimonene, and bismuthene, which differentiates them from other 2D materials, is their colorful allotropes. The additional allotropic forms of black phosphorene, for example, the newly efficiently produced blue phosphorene, are metastable phases. Significantly, buckled *β* allotropes have the lowermost energy, and black phosphorene allotrope has excellent stability. Phosphorene, arsenene, antimonene, and bismuthine allotropes can be synthesized by various approaches. Moreover, the average binding energy of three allotropes discovered in bismuthene, a, b, and z, are comparable. These 2D group VA single‐layered allotropic forms with various energy band placements could help designers create hetero, hybrid, and nanostructured materials, which are critical for new electronic and optoelectronic applications.

Besides the superb fundamental characteristics and wide range of 2D group VA materials, the key difficulty is the deficiency of a cheap and environmental‐benign synthesis approach for large‐scale manufacture. For the applications of transistors, photodetectors, sensors, lithium and sodium batteries, good quality and broad surface area would be established to enable more powerful practical devices. Moreover, the broad range of various allotropic forms of 2D group VA family members make them ideal for diverse potential applications. The 2D group VA members ranging from monolayered structure to heterostructure, provide an innovative foundation for studying device physics in these 2D systems and developing new nanodevice principles.

It is feasible to conclude that 2D structures of group VA components represent a class of materials with extensive theoretical and experimental research potential. All theoretically predicted effects require the fabrication/synthesis of the structures under consideration. We anticipate that the development of novel 2D group VA materials, as well as their functionalization, could provide new challenges. The strategic preparation of 2D group VA materials, including morphology, composition, and tunable surface properties, is critical. There is no conventional method for synthesizing 2D group VA materials, and preparing these materials on a large scale remains a major issue. Recent experimental attempts to obtain few‐layers or monolayers of group VA structures via exfoliation revealed that only phosphorene can be obtained via mechanical exfoliation, while other group VA structures can only be synthesized on substrates via epitaxial growth techniques. As a result, a more profound understanding must be pursued in the future.

For future research, there is an open field for critical features that need to be researched further. While the electrical and optical characteristics of multilayers are affected by the stacking sequence and can be controlled by the number of layers, the nature of interlayer interaction in multilayers is not well known. Multilayers of certain group VA elements may differ from van der Waals solids due to strong chemical interaction between the layers. In this regard, the strength of the chemical interaction between the layers must be thoroughly examined to determine whether vertical connections could develop. The lateral and vertical heterostructure of group VA monolayers are another important research direction that should be focused in the future. While group VA/graphene and group VA/metal heterostructures are being thoroughly investigated, single and multiple heterostructures formed by junctions of different group VA monolayers or multilayers can be a fruitful subject of research with interesting applications. The heterostructures produced by the junction of topologically nontrivial/trivial monolayers will be of significant interest. Although several theoretical investigations demonstrate the energetically or dynamically stable vertical group VA heterostructures, the real applications are still in progress. In addition, certain 2D monolayers of group VA elements can exhibit considerable directionality in a wide range of physical properties. Such an effect could be reproduced in in‐plane, corresponding lateral heterostructures to generate 2D monolayers composed of parallel strips of group VA components. These monolayer or multilayer structures may also have fascinating technological uses.

## 8 Conclusions

This review article intends to provide a clear picture of the 2D group VA materials, including their structures of crystal lattices, basic functions, electrical modulation methods, fabrication techniques, and promising applications. We hope it will spark more rich features and implementations in the expanding family of 2D group VA members.

A reconsideration of group VA materials has motivated the early victory of 2D type to hasten the development of materials that complement the characteristics of their bulk van der Waals solids. 2D layered group VA monoelemental nanomaterials, in contrast to group IVA (semimetallic) and group IIIA (metallic) materials, are semiconductors with a wide variety of bandgaps that can be adjusted, making them perfect candidates for advanced nanodevices. Scientists have indeed been encouraged by recent substantial successes in producing 2D group VA materials to speed up the discovery of novel 2D materials with a wide range of intriguing physicochemical properties, functional designs, and important applications. High stability, higher charge carrier mobility, tunable direct bandgap, and distinctive in‐plane anisotropic structural morphology distinguish 2D group VA monoelemental nanomaterials from other materials. They are better candidates for effective applications in various research areas.

The top‐down and bottom‐up synthetic approaches for 2D group VA materials have been discussed. Various perspectives offer different advantages to diverse research goals. The production of 2D group VA materials on an industrial level with excellent oxidation and degrading resilience is particularly important for improving their physiochemical properties and a wide range of real‐world applications. A combination of diverse processes is an effective strategy for preparing high‐quality 2D group VA materials. Bottom‐up approaches including MBE and CVD are efficient in making 2D group VA monoelemental materials. Solvothermal synthesis and high‐temperature melting can synthesize P, As, Sb, and Bi‐containing hybrids. Using two bottom‐up approaches together can help to create novel 2D group VA heterostructured or/and hybrid materials with exceptional structural features. Organic ligands can help improve product stability by assisting in the preparation process. Future research should therefore concentrate on the investigation and refinement of synthetic methods, focusing on affordable and environment‐friendly acceptable raw materials, easy operation, low energy consumption, and high production rate.

We have investigated the mechanical, optical, and electronic properties. Future studies will need to unravel electron structure‐related mechanisms for the progressive recovery of the current characteristic features of 2D group VA materials and expand these features to other areas. Exploring novel 2D group VA materials on the nanoscale is anticipated to obtain innovative and exceptional characteristics and requires a combination of theoretical models and real investigations. Some important features of 2D group VA materials, such as photothermal effects and upconversion luminescence capabilities, are rarely described.

Moreover, the rapid growth of other 2D materials family members like graphene, TMDs, metal‐organic frameworks, and MXenes will drive future research. 2D group VA nanostructures would be additionally manipulated to provide a wide range of useful nanostructures for various applications. 2D group VA nanostructures primarily encompass synthetic heterostructures, doping of heteroatom (N, P, and S), interface functionalization, and hybrid materials. Importantly, emerging hybrid materials can have innovative and better physicochemical characteristics and functions of the synergistic interactions between nanostructures and other low‐dimensional nanostructured materials. There have been few investigations of functional nanomaterials composed of 2D group VA nanomaterials and 0D/1D materials. In upcoming studies, group VA nanomaterials will be used to cleverly design and fabricate various unique and bifunctional nanostructured materials, including hybrids, 2D‐0D heterostructures, and 2D‐1D heterostructures. These recently developed hybrid composites are anticipated to display innovative and synergistic effects and improved physicochemical characteristics for use in advanced nanodevices and intelligent systems.

2D group VA materials have also provided a variety of significant applications in well‐known research areas, including FETs, memristors, sensors, photodetectors, and batteries. These applications are investigated using theoretical and experimental methods. However, compared to normal 2D systems, the real‐world performance stability and device competency of these systems still need to be enhanced. It is possible to accomplish the excellent characteristics of 2D group VA nanomaterials by enhancing their functional structures and synthetic processes. Scientific investigations should then concentrate on logical design strategies and ideal fabrication techniques using 2D group VA nanosystems for complex functional systems. Applications for the enhanced systems and devices in current fields look promising due to their high stability and efficiency. In addition, it is believed that 2D group VA semiconductors will have new possibilities for implementation in other considerable and comprehensive disciplines, such as laser therapy, biomedical applications, electrical and chemical sensing, versatile and wearable technology, intelligent electronics, and adaptable optoelectronic devices.

## Conflict of Interest

The authors declare no conflict of interest.

## References

[1] a) M. Lanza , Q. Smets , C. Huyghebaert , L.‐J. Li , Nat. Commun. 2020, 11, 5689; 33173041 10.1038/s41467-020-19053-9 PMC7655834

[2] a) J. Qiao , X. Kong , Z.‐X. Hu , F. Yang , W. Ji , Nat. Commun. 2014, 5, 4475; 25042376 10.1038/ncomms5475 PMC4109013

[3] a) S. Zhang , Z. Yan , Y. Li , Z. Chen , H. Zeng , Angew. Chem., Int. Ed. 2015, 127, 3155; 10.1002/anie.201411246 25564773

[4] a) H. Liu , A. T. Neal , Z. Zhu , Z. Luo , X. Xu , D. Tománek , P. D. Ye , ACS Nano 2014, 8, 4033; 24655084 10.1021/nn501226z

[5] a) S. Duan , Y. Cui , X. Chen , W. Yi , Y. Liu , X. Liu , Adv. Funct. Mater. 2019, 29, 1904346;

[6] A. Zhao , H. Li , X. Hu , C. Wang , H. Zhang , J. Lu , S. Ruan , Y.‐J. Zeng , J. Phys. D: Appl. Phys. 2020, 53, 293002.

[7] F. Xia , H. Wang , D. Xiao , M. Dubey , A. Ramasubramaniam , Nat. Photonics 2014, 8, 899.

[8] a) A. K. Geim , I. V. Grigorieva , Nat. Commun. 2013, 499, 419; 10.1038/nature12385 23887427

[9] I. Choudhuri , P. Bhauriyal , B. Pathak , Chem. Mater. 2019, 31, 8260.

[10] T. Wang , H. Wang , Z. Kou , W. Liang , X. Luo , F. Verpoort , Y. J. Zeng , H. Zhang , Adv. Funct. Mater. 2020, 30, 2002885.

[11] H. Lu , J. Gao , Z. Hu , X. Shao , RSC Adv. 2016, 6, 102724.

[12] a) M. Dong , C. He , W. Zhang , J. Phys. Chem. C 2017, 121, 22040;

[13] a) M. Amsler , Z. Yao , C. Wolverton , Chem. Mater. 2017, 29, 9819;

[14] J. H. Xu , E. G. Wang , C. S. Ting , W. P. Su , MRS Online Proc. Libr. 1993, 325, 585.

[15] Y. Yoon , K. Ganapathi , S. Salahuddin , Nano Lett. 2011, 11, 3768. 21790188 10.1021/nl2018178

[16] D. Akinwande , N. Petrone , J. Hone , Nat. Commun. 2014, 5, 5678. 25517105 10.1038/ncomms6678

[17] Y. Takao , H. Asahina , A. Morita , J. Phys. Soc. Jpn. 1981, 50, 3362.

[18] a) L. Kou , T. Frauenheim , C. Chen , J. Phys. Chem. Lett. 2014, 5, 2675; 26277962 10.1021/jz501188k

[19] R. Hultgren , N. Gingrich , B. Warren , J. Chem. Phys. 1935, 3, 351.

[20] K. Ashley , D. Cordell , D. Mavinic , Chemosphere 2011, 84, 737. 21481914 10.1016/j.chemosphere.2011.03.001

[21] A. Carvalho , M. Wang , X. Zhu , A. S. Rodin , H. Su , A. H. Castro Neto , Nat. Rev. Mater. 2016, 1, 16061.

[22] L. Zhang , H. Huang , B. Zhang , M. Gu , D. Zhao , X. Zhao , L. Li , J. Zhou , K. Wu , Y. Cheng , Angew. Chem. 2020, 132, 1090. 10.1002/anie.201912761 31713959

[23] L. Kou , C. Chen , S. C. Smith , J. Phys. Chem. Lett. 2015, 6, 2794. 26266865 10.1021/acs.jpclett.5b01094

[24] a) H. Fei , W. Di , C. D. Malliakas , M. Sturza , D. Y. Chung , X. Wan , M. G. Kanatzidis , ChemInform 2015, 46, 3112;

[25] J. Ji , X. Song , J. Liu , Z. Yan , C. Huo , S. Zhang , M. Su , L. Liao , W. Wang , Z. Ni , Nat. Commun. 2016, 7, 13352. 27845327 10.1038/ncomms13352 PMC5116078

[26] Y. Hu , J. Liang , Y. Xia , C. Zhao , M. Jiang , J. Ma , Z. Tie , Z. Jin , Small 2021, 18, 2104556. 10.1002/smll.202104556 34846791

[27] Y. Wang , P. Huang , M. Ye , R. Quhe , Y. Pan , H. Zhang , H. Zhong , J. Shi , J. Lu , Chem. Mater. 2017, 29, 2191.

[28] M. Zeraati , S. M. V. Allaei , I. A. Sarsari , M. Pourfath , D. Donadio , Phys. Rev. B 2016, 93, 085424.

[29] O. Ü. Aktürk , V. O. Özçelik , S. Ciraci , Phys. Rev. B 2015, 91, 235446.

[30] W. Tian , S. Zhang , C. Huo , D. Zhu , Q. Li , L. Wang , X. Ren , L. Xie , S. Guo , P. K. Chu , ACS Nano 2018, 12, 1887. 29370516 10.1021/acsnano.7b08714

[31] W. Lin , Y. Lian , G. Zeng , Y. Chen , Z. Wen , H. Yang , Nano Res. 2018, 11, 5968.

[32] T. Nagao , S. Yaginuma , M. Saito , T. Kogure , J. Sadowski , T. Ohno , S. Hasegawa , T. Sakurai , Surf. Sci. 2005, 590, 247.

[33] a) H. Huang , X. Ren , Z. Li , H. Wang , Z. Huang , H. Qiao , P. Tang , J. Zhao , W. Liang , Y. Ge , Nanotechnology 2018, 29, 235201; 29543188 10.1088/1361-6528/aab6ee

[34] Y. Guo , F. Pan , M. Ye , X. Sun , Y. Wang , J. Li , X. Zhang , H. Zhang , Y. Pan , Z. Song , ACS Appl. Mater. Interfaces 2017, 9, 23128. 28597660 10.1021/acsami.7b03833

[35] E. Aktürk , O. Ü. Aktürk , S. Ciraci , Phys. Rev. B 2016, 94, 014115.

[36] a) T. Chai , X. Li , T. Feng , P. Guo , Y. Song , Y. Chen , H. Zhang , Nanoscale 2018, 10, 17617; 30204206 10.1039/c8nr03068e

[37] F. Wang , R. Tang , H. Yu , P. Gibbons , W. Buhro , S. Louis , Chem. Mater. 2008, 1, 3656.

[38] P. Kumar , J. Singh , A. C. Pandey , RSC Adv. 2013, 3, 2313.

[39] R. Gusmão , Z. Sofer , D. Bouša , M. Pumera , Angew. Chem. 2017, 129, 14609. 10.1002/anie.201706389 28755460

[40] L. Lu , Z. Liang , L. Wu , Y. Chen , Y. Song , S. C. Dhanabalan , J. S. Ponraj , B. Dong , Y. Xiang , F. Xing , Laser Photonics Rev. 2018, 12, 1700221.

[41] J. Wu , H. Yang , H. Li , Z. Lu , X. Yu , R. Chen , J. Alloys Compd. 2010, 498, 8.

[42] F. Reis , G. Li , L. Dudy , M. Bauernfeind , S. Glass , W. Hanke , R. Thomale , J. Schäfer , R. Claessen , Science 2017, 357, 287. 28663438 10.1126/science.aai8142

[43] M. Zhou , W. Ming , Z. Liu , Z. Wang , P. Li , F. Liu , Proc. Natl. Acad. Sci. USA 2014, 111, 14378. 25246584 10.1073/pnas.1409701111 PMC4210051

[44] a) R. Mohan , Nat. Chem. 2010, 2, 336; 21124518 10.1038/nchem.609

[45] Y. Liu , N. O. Weiss , X. Duan , H.‐C. Cheng , Y. Huang , X. Duan , Nat. Rev. Mater. 2016, 1, 16042.

[46] A. Rockett , The Materials Science of Semiconductors, 1st ed., Springer, New York 2008.

[47] J.‐H. Lee , E. K. Lee , W.‐J. Joo , Y. Jang , B.‐S. Kim , J. Y. Lim , S.‐H. Choi , S. J. Ahn , J. R. Ahn , M.‐H. Park , Science 2014, 344, 286. 24700471 10.1126/science.1252268

[48] Y. Chen , G. Jiang , S. Chen , Z. Guo , X. Yu , C. Zhao , H. Zhang , Q. Bao , S. Wen , D. Tang , Opt. Express 2015, 23, 12823. 26074536 10.1364/OE.23.012823

[49] J. Miao , S. Zhang , L. Cai , M. Scherr , C. Wang , ACS Nano 2015, 9, 9236. 26277886 10.1021/acsnano.5b04036

[50] S. Zhang , J. Yang , R. Xu , F. Wang , W. Li , M. Ghufran , Y.‐W. Zhang , Z. Yu , G. Zhang , Q. J. Qin , ACS Nano 2014, 8, 9590. 25188827 10.1021/nn503893j

[51] P. Ares , F. Aguilar‐Galindo , D. Rodríguez‐San‐Miguel , D. A. Aldave , S. Díaz‐Tendero , M. Alcamí , F. Martín , J. Gómez‐Herrero , F. Zamora , Adv. Mater. 2016, 28, 6332. 27272099 10.1002/adma.201602128

[52] R. Gui , H. Jin , Y. Sun , X. Jiang , Z. Sun , J. Mater. Chem. A 2019, 7, 25712.

[53] P. Ares , J. J. Palacios , G. Abellán , J. Gómez‐Herrero , F. Zamora , Adv. Mater. 2018, 30, 1703771. 10.1002/adma.201703771 29076558

[54] C. C. Mayorga‐Martinez , R. Gusmão , Z. Sofer , M. Pumera , Angew. Chem., Int. Ed. 2019, 58, 134. 10.1002/anie.201808846 30421531

[55] R. Gusmão , Z. Sofer , D. Bouša , M. Pumera , Angew. Chem., Int. Ed. 2017, 129, 14609. 10.1002/anie.201706389 28755460

[56] R. B. Jacobs‐Gedrim , M. T. Murphy , F. Yang , N. Jain , M. Shanmugam , E. S. Song , Y. Kandel , P. Hesamaddin , H. Y. Yu , M. Anantram , Appl. Phys. Lett. 2018, 112, 133101.

[57] D. Teweldebrhan , V. Goyal , A. A. Balandin , Nano Lett. 2010, 10, 1209. 20205455 10.1021/nl903590b

[58] Y. Xu , H. Cao , Y. Xue , B. Li , W. Cai , Nanomaterials 2018, 8, 942. 30445778 10.3390/nano8110942 PMC6265730

[59] a) D. Hanlon , C. Backes , E. Doherty , C. S. Cucinotta , N. C. Berner , C. Boland , K. Lee , A. Harvey , P. Lynch , Z. Gholamvand , Nat. Commun. 2015, 6, 8563; 26469634 10.1038/ncomms9563 PMC4634220

[60] W. Guo , H. Song , S. Yan , Opt. Commun. 2018, 406, 91.

[61] P. Vishnoi , M. Mazumder , S. K. Pati , C. R. Rao , New J. Chem. 2018, 42, 14091.

[62] C. Gibaja , D. Rodriguez‐San‐Miguel , P. Ares , J. Gómez‐Herrero , M. Varela , R. Gillen , J. Maultzsch , F. Hauke , A. Hirsch , G. Abellán , Angew. Chem., Int. Ed. 2016, 128, 14557. 10.1002/anie.201605298 PMC5113666 27529687

[63] W. Tao , X. Ji , X. Xu , M. A. Islam , Z. Li , S. Chen , P. E. Saw , H. Zhang , Z. Bharwani , Z. Guo , Angew. Chem. 2017, 129, 12058. 10.1002/anie.201703657 PMC5608550 28640986

[64] C. Xing , W. Huang , Z. Xie , J. Zhao , D. Ma , T. Fan , W. Liang , Y. Ge , B. Dong , J. Li , ACS Photonics 2018, 5, 621.

[65] T. Fan , Z. Xie , W. Huang , Z. Li , H. Zhang , Nanotechnology 2019, 30, 114002. 30609416 10.1088/1361-6528/aafc0f

[66] Z. Xie , C. Xing , W. Huang , T. Fan , Z. Li , J. Zhao , Y. Xiang , Z. Guo , J. Li , Z. Yang , Adv. Funct. Mater. 2018, 28, 1705833.

[67] X. Ge , Z. Xia , S. Guo , Adv. Funct. Mater. 2019, 29, 1900318.

[68] X. Wang , J. He , B. Zhou , Y. Zhang , J. Wu , R. Hu , L. Liu , J. Song , J. Qu , Angew. Chem., Int. Ed. 2018, 130, 8804. 10.1002/anie.201804886 29786932

[69] J. R. Brent , N. Savjani , E. A. Lewis , S. J. Haigh , D. J. Lewis , P. O'Brien , Chem. Commun. 2014, 50, 13338. 10.1039/c4cc05752j 25231502

[70] Z. J. Han , A. T. Murdock , D. H. Seo , A. Bendavid , 2D Mater. 2018, 5, 032002.

[71] V. M. Donnelly , A. Kornblit , J. Vac. Sci. Technol., A 2013, 31, 050825.

[72] W. Gao , Z. Zheng , P. Wen , N. Huo , J. Li , Nanophotonics 2020, 9, 2147.

[73] J. Pei , X. Gai , J. Yang , X. Wang , Z. Yu , D.‐Y. Choi , B. Luther‐Davies , Y. Lu , Nat. Commun. 2016, 7, 10450. 26794866 10.1038/ncomms10450 PMC4735856

[74] D. Kong , W. Dang , J. J. Cha , H. Li , S. Meister , H. Peng , Z. Liu , Y. Cui , Nano Lett. 2010, 10, 2245. 20486680 10.1021/nl101260j

[75] N. Hussain , T. Liang , Q. Zhang , T. Anwar , Y. Huang , J. Lang , K. Huang , H. Wu , Small 2017, 13, 1701349. 10.1002/smll.201701349 28762634

[76] a) N. Liu , P. Kim , J. H. Kim , J. H. Ye , S. Kim , C. J. Lee , ACS Nano 2014, 8, 6902; 24937086 10.1021/nn5016242

[77] P. Yu , S. E. Lowe , G. P. Simon , Y. L. Zhong , Curr. Opin. Colloid Interface Sci 2015, 20, 329.

[78] L. Lu , X. Tang , R. Cao , L. Wu , Z. Li , G. Jing , B. Dong , S. Lu , Y. Li , Y. Xiang , Adv. Opt. Mater. 2017, 5, 1700301.

[79] L. Sun , G. Yuan , L. Gao , J. Yang , M. Chhowalla , M. H. Gharahcheshmeh , K. K. Gleason , Y. S. Choi , B. H. Hong , Z. Liu , Nat. Rev. Methods Primers 2021, 1, 5.

[80] M. Zhao , J. Su , Y. Zhao , P. Luo , F. Wang , W. Han , Y. Li , X. Zu , L. Qiao , T. Zhai , Adv. Funct. Mater. 2020, 30, 1909849.

[81] F. Yang , R. B. Jacobs‐Gedrim , M. Shanmugam , N. Jain , M. T. Murphy , E. S. Song , D. Frey , B. Yu , RSC Adv. 2015, 5, 59320.

[82] J. Gao , L. Zhang , W. Liu , S. Li , Mater. Lett. 2020, 268, 127588.

[83] H. C. Chang , C. L. Tu , K. I. Lin , J. Pu , T. Takenobu , C. N. Hsiao , C. H. Chen , Small 2018, 14, 1802351. 10.1002/smll.201802351 30152600

[84] J. You , M. D. Hossain , Z. Luo , Nano Convergence 2018, 5, 26. 30467647 10.1186/s40580-018-0158-x PMC6160381

[85] Z. Cai , B. Liu , X. Zou , H.‐M. Cheng , Chem. Rev. 2018, 118, 6091. 29384374 10.1021/acs.chemrev.7b00536

[86] Q. Wang , M. Safdar , K. Xu , M. Mirza , Z. Wang , J. He , ACS Nano 2014, 8, 7497. 24988364 10.1021/nn5028104

[87] J. L. Zhang , S. Zhao , C. Han , Z. Wang , S. Zhong , S. Sun , R. Guo , X. Zhou , C. D. Gu , K. D. Yuan , Nano Lett. 2016, 16, 4903. 27359041 10.1021/acs.nanolett.6b01459

[88] Z. Zhu , D. Tománek , Phys. Rev. Lett. 2014, 112, 176802. 24836265 10.1103/PhysRevLett.112.176802

[89] X. Wu , Y. Shao , H. Liu , Z. Feng , Y. L. Wang , J. T. Sun , C. Liu , J. O. Wang , Z. L. Liu , S. Y. Zhu , Adv. Mater. 2017, 29, 1605407. 10.1002/adma.201605407 28028843

[90] T. Nagao , J. Sadowski , M. Saito , S. Yaginuma , Y. Fujikawa , T. Kogure , T. Ohno , Y. Hasegawa , S. Hasegawa , T. Sakurai , Phys. Rev. Lett. 2004, 93, 105501. 15447414 10.1103/PhysRevLett.93.105501

[91] E. S. Walker , S. R. Na , D. Jung , S. D. March , J.‐S. Kim , T. Trivedi , W. Li , L. Tao , M. L. Lee , K. M. Liechti , Nano Lett. 2016, 16, 6931. 27775368 10.1021/acs.nanolett.6b02931

[92] J. Chen , Y. Dai , Y. Ma , X. Dai , W. Ho , M. Xie , RSC Nanoscale 2017, 9, 15945. 10.1039/c7nr04085g 29019513

[93] Y. Zhang , X. Rui , Y. Tang , Y. Liu , J. Wei , S. Chen , W. R. Leow , W. Li , Y. Liu , J. Deng , Adv. Energy Mater. 2016, 6, 1502409.

[94] B. Tian , B. Tian , B. Smith , M. Scott , Q. Lei , R. Hua , Y. Tian , Y. Liu , Proc. Natl. Acad. Sci. USA 2018, 115, 4345. 29563225 10.1073/pnas.1800069115 PMC5924925

[95] M. Amani , C. Tan , G. Zhang , C. Zhao , J. Bullock , X. Song , H. Kim , V. R. Shrestha , Y. Gao , K. B. Crozier , ACS Nano 2018, 12, 7253. 29912549 10.1021/acsnano.8b03424

[96] D. Xiang , C. Han , J. Wu , S. Zhong , Y. Liu , J. Lin , X. A. Zhang , W. P. Hu , B. zyilmaz , A. Neto , Nat. Commun. 2015, 6, 6485. 25761440 10.1038/ncomms7485

[97] Y. Pan , Y. Wang , M. Ye , R. Quhe , H. Zhong , Z. Song , X. Peng , D. Yu , J. Yang , J. Shi , Chem. Mater. 2015, 27, 1707.

[98] M. V. Kamalakar , B. N. Madhushankar , A. Dankert , S. P. Dash , Small 2015, 11, 2209. 25586013 10.1002/smll.201402900

[99] W. Michailow , P. Spencer , N. W. Almond , S. J. Kindness , R. Wallis , T. A. Mitchell , R. Degl'Innocenti , S. A. Mikhailov , H. E. Beere , D. A. Ritchie , Sci. Adv. 2022, 8, 8398. 10.1126/sciadv.abi8398 PMC9012455 35427162

[100] a) X. Ren , Z. Li , Z. Huang , D. Sang , H. Qiao , X. Qi , J. Li , J. Zhong , H. Zhang , Adv. Funct. Mater. 2017, 27, 1606834;

[101] Q. Xiao , C. X. Hu , H. R. Wu , Y. Y. Ren , X. Y. Li , Q. Yang , G. H. Dun , Z. P. Huang , Y. Peng , F. Yan , Nanoscale Horiz. 2019, 5, 124.

[102] Y. Liu , B. N. Shivananju , Y. Wang , Y. Zhang , W. Yu , S. Xiao , T. Sun , W. Ma , H. Mu , S. Lin , ACS Appl. Mater. Interfaces 2017, 9, 36137. 28948769 10.1021/acsami.7b09889

[103] F. Wang , Z. Wang , T. A. Shifa , Y. Wen , F. Wang , X. Zhan , Q. Wang , K. Xu , Y. Huang , L. Yin , Adv. Funct. Mater. 2017, 27, 1603254.

[104] a) T. Xian , W. Liang , J. Zhao , Z. Li , Z. Han , Small 2017, 13, 1702739;

[105] C. Zhang , W. B. Ye , K. Zhou , H. Y. Chen , J. Q. Yang , G. Ding , X. Chen , Y. Zhou , L. Zhou , F. Li , Adv. Funct. Mater. 2019, 29, 1808783.

[106] J. Y. Mao , S. Wu , G. Ding , Z. P. Wang , F. S. Qian , J. Q. Yang , Y. Zhou , S. T. Han , Small 2022, 18, 2106253. 10.1002/smll.202106253 35083839

[107] V. K. Perla , S. K. Ghosh , K. Mallick , J. Mater. Chem. C 2021, 9, 6904.

[108] S. T. Han , H. Peng , Q. Sun , S. Venkatesh , K. S. Chung , S. C. Lau , Y. Zhou , V. Roy , Adv. Mater. 2017, 29, 1700375. 10.1002/adma.201700375 28671711

[109] T. Xue , W. Liang , Y. Li , Y. Sun , Y. Xiang , Y. Zhang , Z. Dai , Y. Duo , L. Wu , K. Qi , Nat. Commun. 2019, 10, 28. 30604756 10.1038/s41467-018-07947-8 PMC6318270

[110] Y. Jing , L. B. Chang , C. Jy , Comput. Theor. Chem. 2020, 1190, 112977.

[111] a) L. Li , L. Chen , S. Mukherjee , J. Gao , H. Sun , Z. Liu , X. Ma , T. Gupta , C. V. Singh , W. Ren , Adv. Mater. 2017, 29, 1602734; 10.1002/adma.201602734 27797133

[112] J.‐M. Yang , E.‐S. Choi , S.‐Y. Kim , J.‐H. Kim , J.‐H. Park , N.‐G. Park , Nanoscale 2019, 11, 6453. 30892306 10.1039/c8nr09918a

[113] J. Zhou , J. Chen , M. Chen , J. Wang , X. Liu , B. Wei , Z. Wang , J. Li , L. Gu , Q. Zhang , Adv. Mater. 2019, 31, 1807874. 10.1002/adma.201807874 30714223

[114] Y. Wu , W. Bensch , J Alloys Compd. 2011, 509, 4452.

[115] W. Huang , C. Xing , Y. Wang , Z. Li , L. Wu , D. Ma , X. Dai , Y. Xiang , J. Li , D. Fan , Nanoscale 2018, 10, 2404. 29334393 10.1039/c7nr09046c

[116] J. Yao , K. J. Koski , W. Luo , J. J. Cha , L. Hu , D. Kong , V. K. Narasimhan , K. Huo , Y. Cui , Nat. Commun. 2014, 5, 5670. 25430612 10.1038/ncomms6670

[117] G. Abellán , P. Ares , S. Wild , E. Nuin , C. Neiss , D. R. S. Miguel , P. Segovia , C. Gibaja , E. G. Michel , A. Görling , Angew. Chem., Int. Ed. 2017, 56, 14389. 10.1002/anie.201702983 PMC5698728 28945952

[118] Y. Song , Y. Chen , X. Jiang , W. Liang , K. Wang , Z. Liang , Y. Ge , F. Zhang , L. Wu , J. Zheng , Adv. Opt. Mater. 2018, 6, 1701287.

[119] Y. Xu , Z. Wang , Z. Guo , H. Huang , Q. Xiao , H. Zhang , X.‐F. Yu , Adv. Opt. Mater. 2016, 4, 1223.

[120] Y. Shao , Z.‐L. Liu , C. Cheng , X. Wu , H. Liu , C. Liu , J.‐O. Wang , S.‐Y. Zhu , Y.‐Q. Wang , D.‐X. Shi , Nano Lett. 2018, 18, 2133. 29457727 10.1021/acs.nanolett.8b00429

[121] T. Märkl , P. J. Kowalczyk , M. L.e Ster , I. V. Mahajan , H. Pirie , Z. Ahmed , G. Bian , X. Wang , T.‐C. Chiang , S. A. Brown , 2D Mater. 2017, 5, 011002.

[122] H.‐S. Tsai , S.‐W. Wang , C.‐H. Hsiao , C.‐W. Chen , H. Ouyang , Y.‐L. Chueh , H.‐C. Kuo , J.‐H. Liang , Chem. Mater. 2016, 28, 425.

[123] A. Ambrosi , Z. Sofer , M. Pumera , Angew. Chem. 2017, 129, 10579. 10.1002/anie.201705071 28649798

[124] B. Wang , Y. Zhou , Z. Huang , H. Qiao , C. Duan , X. Ren , Z. Wang , J. Zhong , X. Qi , Mater. Today: Proc. 2021, 14, 100109.

[125] J. Wang , S. U. Rehman , Y. Xu , B. Zuo , H. Cheng , L. Guo , B. Zou , X. Zhang , C. Li , Sol. Energy 2022, 233, 213.

